# The Prognostic Significance of Metabolic Response Heterogeneity in Metastatic Colorectal Cancer

**DOI:** 10.1371/journal.pone.0138341

**Published:** 2015-09-30

**Authors:** Alain Hendlisz, Amelie Deleporte, Thierry Delaunoit, Raphaël Maréchal, Marc Peeters, Stéphane Holbrechts, Marc Van den Eynde, Ghislain Houbiers, Bertrand Filleul, Jean-Luc Van Laethem, Sarah Ceyssens, Anna-Maria Barbuto, Renaud Lhommel, Gauthier Demolin, Camilo Garcia, Hazem El Mansy, Lieveke Ameye, Michel Moreau, Thomas Guiot, Marianne Paesmans, Martine Piccart, Patrick Flamen

**Affiliations:** 1 Medical Oncology Department, Institut Jules Bordet, Université Libre de Bruxelles, Brussels, Belgium; 2 Oncology Department, Jolimont Hospital, Haine-St-Paul, Belgium; 3 Gastroenterology Medico-Surgical Department, Erasme University Hospital, Université Libre de Bruxelles, Brussels, Belgium; 4 Oncology Department, Antwerp University Hospital, Antwerp University, Edegem, Belgium; 5 Nuclear Medicine Department, Antwerp, University Hospital, Edegem, Belgium; 6 Oncology Department, CHU Ambroise Pare, Mons, Belgium; 7 Oncology Department, Cliniques Universitaires Saint-Luc, Université Catholique de Louvain, Brussels, Belgium; 8 Nuclear Medicine Department, Cliniques Universitaires Saint-Luc, Université Catholique de Louvain, Brussels, Belgium; 9 Gastroenterology Department, CHC ST Joseph, Liege, Belgium; 10 Nuclear Medicine Department, Institut Jules Bordet, Université Libre de Bruxelles, Brussels, Belgium; 11 Data centre, Institut Jules Bordet, Université Libre de Bruxelles, Brussels, Belgium; University Campus Bio-Medico, ITALY

## Abstract

**Background:**

Tumoral heterogeneity is a major determinant of resistance in solid tumors. FDG-PET/CT can identify early during chemotherapy non-responsive lesions within the whole body tumor load. This prospective multicentric proof-of-concept study explores intra-individual metabolic response (mR) heterogeneity as a treatment efficacy biomarker in chemorefractory metastatic colorectal cancer (mCRC).

**Methods:**

Standardized FDG-PET/CT was performed at baseline and after the first cycle of combined sorafenib (600mg/day for 21 days, then 800mg/day) and capecitabine (1700 mg/m²/day administered D1-14 every 21 days). MR assessment was categorized according to the proportion of metabolically non-responding (non-mR) lesions (stable FDG uptake with SUVmax decrease <15%) among all measurable lesions.

**Results:**

Ninety-two patients were included. The median overall survival (OS) and progression-free survival (PFS) were 8.2 months (95% CI: 6.8–10.5) and 4.2 months (95% CI: 3.4–4.8) respectively. In the 79 assessable patients, early PET-CT showed no metabolically refractory lesion in 47%, a heterogeneous mR with at least one non-mR lesion in 32%, and a consistent non-mR or early disease progression in 21%. On exploratory analysis, patients without any non-mR lesion showed a significantly longer PFS (HR 0.34; 95% CI: 0.21–0.56, P-value <0.001) and OS (HR 0.58; 95% CI: 0.36–0.92, P-value 0.02) compared to the other patients. The proportion of non-mR lesions within the tumor load did not impact PFS/OS.

**Conclusion:**

The presence of at least one metabolically refractory lesion is associated with a poorer outcome in advanced mCRC patients treated with combined sorafenib-capecitabine. Early detection of treatment-induced mR heterogeneity may represent an important predictive efficacy biomarker in mCRC.

**Trial Registration:**

ClinicalTrials.gov NCT01290926

## Introduction

The development of new therapeutics for solid tumors is currently strained by increasing regulatory demands to better define subpopulations bearing resistant diseases in order to spare patients from useless toxicities and the society from unaffordable costs in case of ineffective treatments.

Tumor heterogeneity through the existence of resistant subclones (genetic drift) or local environmental factors is nowadays accepted as a major determinant of treatment resistance. However, sensitive biomarkers of tumoral heterogeneity are lacking.[1–3] Current response assessment methods using morphology (RECIST using MRI/CT) or metabolism (PERCIST using FGD-PET/CT) do not allow the description of tumor heterogeneity because dichotomization of response (versus non-response) requires summing of measurements or the selection of the one single most representative lesion.[4] Moreover most of the new biological therapies render response evaluation even more challenging by the infrequency of tumor shrinkage.[5–8]

Imaging tumour metabolism using ^18^F-Fluorodeoxyglucose positron emission tomography coupled with computed tomography (FDG-PET/CT) allows rapid identification of treatment-refractory lesions with a high negative predictive value (NPV).[9–14] FDG-PET is currently central in the international recommendations for response assessment for Hodgkin’s disease and aggressive non-Hodgkin’s lymphoma, in which medical conditions it is used commonly as a basis for therapeutic decisions. [14–17] In contrast, solid tumors are frequently more refractory to treatment and reveal smaller and slower changes in FDG uptake under therapy leading to the existence of different criteria for metabolic response assessment at the lesion as well as at the patient level.[18,19] This ongoing discussion explain why metabolic imaging has still not acquired a biomarker status in solid tumors.

Metabolic imaging provides a whole-body quantitative assessment of treatment-induced changes in tumoral glycolysis early after treatment initiation, before any morphological changes are observed. It has therefore the potential to detect tumoral heterogeneity by revealing how distinct tumor sites behave in response to treatment.

Several trials suggest meaningful clinical activity of combined sorafenib-capecitabine in metastatic breast and colorectal cancer. However the significant toxicity of the combination renders its use practically incompatible with a palliative setting, further underscoring the need to identify a sensitive biomarker for patient selection.[20,21] Preliminary reports in lung and renal cancer suggest that FDG-PET-based metabolic response assessment could be used as a predictive biomarker of sorafenib.[22,23]

The trial is a proof-of-concept study designed to explore intra-individual mR heterogeneity as a prognostic biomarker for this combination of a biological and a cytotoxic agent in mCRC.

## Material and Methods

Belgian competent authorities and ethical committees of the 6 participating centres approved the study (EudraCT 2010-023695-91, clinicaltrials.gov NCT01290926), designed as a prospective multicentric single-arm phase II, with one-stage accrual.

Patients with histologically proven unresectable metastatic CRC failing all standard treatments but not necessarily bevacizumab were eligible. Exclusion criteria were contraindications for capecitabine and sorafenib, ECOG performance status (PS) > 1, age < 18 years, and cerebral metastasis. Normal organ and bone marrow function, a life expectancy >12 weeks, and a signed informed consent were required.

Both drugs were given orally on an outpatient basis: sorafenib 200mg in the morning and 400 mg in the evening every day for the first cycle, then 400 mg twice a day every day; capecitabine 850 mg/m^2^ twice a day on days 1 to 14, every 21 days. One cycle was defined as a 21-day period. Adverse events were reported according to the National Cancer Institute Criteria, version 3.0 (http://ctep.cancer.gov/protocolDevelopment/electronic_applications/docs/ctcaev3.pdf). Study medications were to be stopped at disease progression or when unacceptable toxicity occurred. RECIST 1.1-radiological response was assessed locally every two cycles (6weeks). Patients were followed until objective disease progression and every 3 months thereafter for survival assessment.

### FDG-PET/CT Imaging

For the FDG-PET/CT, patients were referred to one of the 5 participating PET/CT centres, previously approved for participation based on FDG-PET phantom imaging study for quality’s central assessment [24]. An independent academic molecular imaging core laboratory (OriLab) centralized all FDG-PET/CT images through anonymized CD-Rom transfers, checked image’s quality, DICOM headers, compliance to the Standard Procedures Imaging Manual and imaging case report forms.

Baseline FDG-PET/CT was performed within 7 days preceding chemotherapy initiation and repeated under the same technical and patient conditions on day (D)21 (range D19-D23), with D1 as the first day of chemotherapy administration. Prior to FDG injection, fasting ≥ 6 hours and glycemia levels <120 mg/dL for non-diabetic patients, and <180 mg/dL for diabetic patients were required. Short-acting insulin use on the day of FDG-PET/CT was not allowed.

The PET/CT was initiated 60 to 90 minutes after intravenous injection of 3.7 to 7.4 MBq/kg FDG, optimized for body weight. Similar FDG activity (+/-15%) and time window (+/- 15 min) were used for the second PET/CT.

Whole body scanning with a low dose CT (without intravenous or oral contrast, from proximal femur to skull) was performed, immediately followed by the PET acquisition. Imaging acquisition and reconstruction remained stable over the whole study period. The second FDG-PET/CT was strictly blinded to the investigators, and was not added to the patient’s (electronic) medical records.

The standard uptake value (SUV) of FDG used was the lean body mass-based maximal SUV value within the lesion (SUVmax, g/ml).

**Fig 1:**
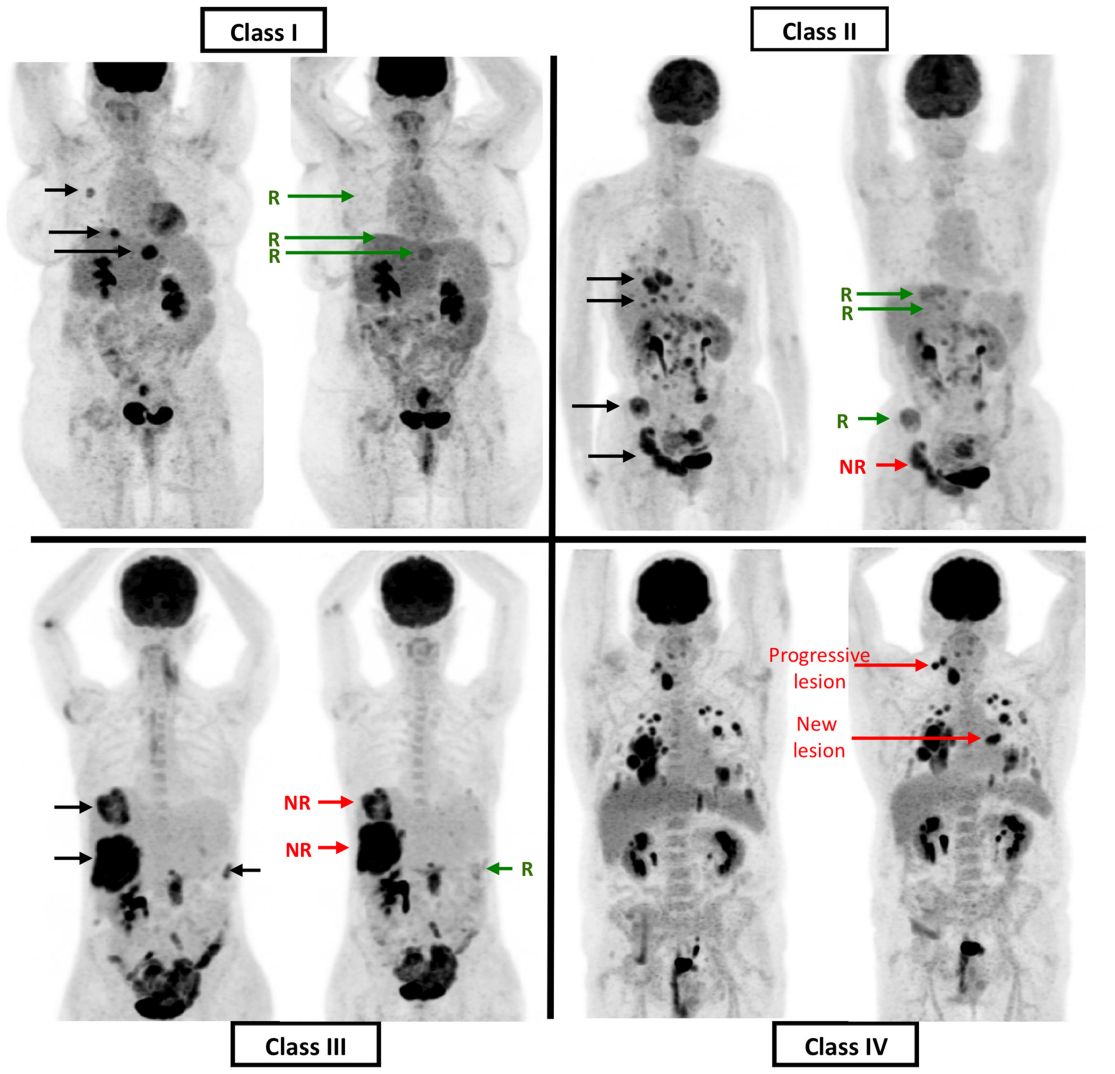
Classes of metabolic responses. Class 1: no metabolic unresponsive lesion; Class 2: minority of unresponsive lesion among whole body target tumour load; Class 3: majority of whole body target tumour load does not respond; Class 4: all target lesions are non-responding, or, presence of progressive lesions [progression defined as >25% increase of FDG uptake on second PET, or appearance of a new lesion].

All FDG-PET/CT images were analysed in batches using the same software (PETVcar version 4.6, General Electric, USA) and display techniques. Two senior nuclear medicine physicians (PF, CG) performed independent mR analyses using a predefined 3-step methodology.[13] First, on the baseline PET/CT, target lesions were identified according to the following criteria: transaxial diameter (measured on the CT of the PET/CT) > 15 mm, intense FDG uptake (> 2 x normal liver parenchym uptake) and with an unequivocally neoplastic basis. Each target lesion was then classified as non-responding (decrease of SUVmax on second PET-CT<15%) or responding. Second, the patients were classified according to the lesional distribution of mR; class I: absence of any metabolically non-responding lesion, class II: a minor part of whole body tumour load shows a non-response, class III: major part of whole body target tumour load does not respond, and, class IV: all target lesions are non-responding, or presence of a progressive lesion (progression defined as >25% increase of SUVMax, or appearance of a new lesion). (Fig 1) Finally, different methods of patient response dichotomization (metabolic responders versus non-responders) were explored.

### Statistical considerations

A first co-primary objective defined the minimal clinical activity necessary to explore the negative predictive value of metabolic response imaging on OS as a survival rate at 6 months > 30% according to the existing literature on chemorefractory CRC. To reject the null hypothesis that the 6 month-OS rate would be <30% using a binomial distribution, a 1-sided test with α = 0.025 and a power of 90% in case of a true 6 months-OS ≥ 50% was used, requiring a sample size of 66 eligible patients followed for at least 6 months. An intent-to-treat (ITT) approach was used.

The second co-primary objective was the prognostic value of mR classification. Based on a previous study,[13] and anticipating a 95% eligibility rate, a 50% early PET/CT non-responders rate, and a hazard ratio (HR) around 0.385 for comparison between the survival distributions, 54 events were needed for a 90% power and a two-sided logrank test at the 2.5% level.

Because the mR rate monitored during the study was higher than expected, the number of events to be observed was increased to 62. This decision was taken without changing the HR to be detected and without estimating this HR during study conduct.

Secondary objectives were to describe PFS, objective response rate and toxicity and to determine the predictive value of early MR on PFS.

For the first co-primary objective, the 6 month-OS, median (m)OS and mPFS were calculated from the patient’s inclusion. For the second co-primary objective, the predictive value assessment of mR on OS and PFS was done from the time of the second FDG-PET/CT on patients having undergone the second FDG-PET/CT in order to control for guarantee-time bias.[25] PFS was calculated up to the time of disease progression or death, whichever occurred first. Kaplan-Meier estimates were used to characterise PFS and OS, and the log-rank test to investigate comparisons between survival curves. Cox’s proportional hazards model was used to calculate HR and their 95% CI

The multivariate analysis was performed using Cox’s proportional hazard model. Variables with a univariate P-value < 0.20 were considered as possible predictors in the multivariate model. We performed stepwise forward selection of variables, i.e. forward selection but at each step variables already in the model could be dropped if their associated p-value became >0.05. To verify the final model, also backward selection of variables was performed on all variables with univariate p-value<0.20, resulting in the same set of variables.[26]

All statistical analyses were performed using SAS 9.4 (SAS Institute Inc., Cary, NC, USA) and GraphPad Prism 6 software.

Patients found with an early metabolic progressive disease (class IV) were not excluded from the statistical analyses as the objectives of the paper were to show the predictive value of early metabolic response on OS and PFS, which implies the necessity of an intent-to-treat analysis. The event “progression” in the definition of PFS is moreover a radiological progression. Patients belonging to class IV do not meet this definition of radiological progression, which remains an event to be predicted.

## Results

**Fig 2:**
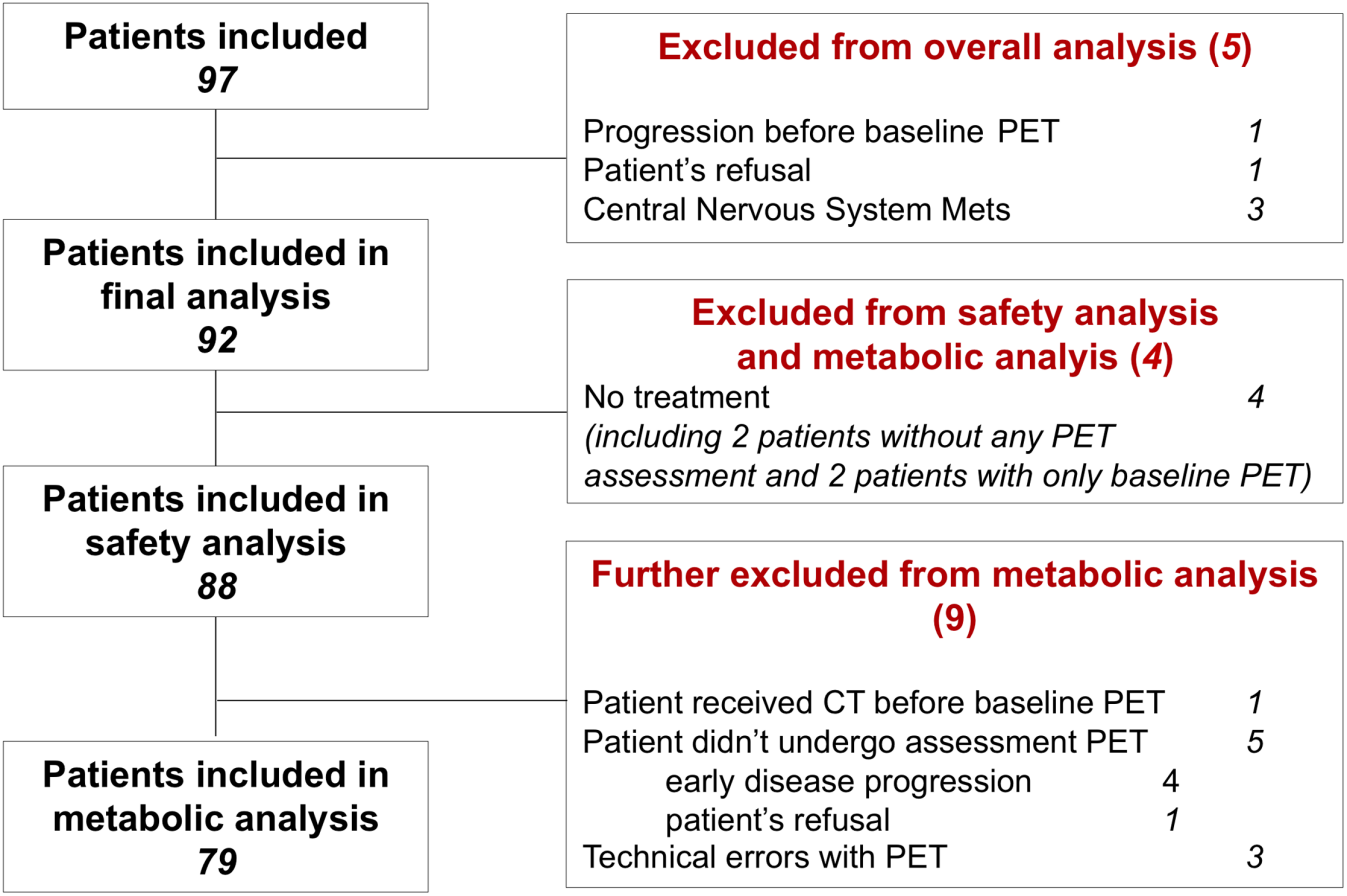
Consort Diagram.

Between February and October 2011, 97 consecutive patients were enrolled in 6 clinical centres. The CONSORT diagram details the reasons for considering 5 patients as ineligible, excluding them from all analysis (Fig 2). The eligible patients (*N* = 92), median age 63 (range 28–83), male/female ratio of 54/46, PS 0 (55%) or 1(45%) received a median of 5 (range 0–44+) cycles of sorafenib-capecitabine after an history of a median of 3 (range 1–6) prior therapeutic lines including bevacizumab in 55% of patients. Codons 12–13 KRAS mutations were present in 52%.

### Toxicity (Table 1)

**Table 1:** Most important (>10%) side effects in the 88 patients who received treatment according to Common Toxicity Criteria CTC3.0.

Adverse Event	All grades (%)	Grade I-II (%)	Grade III-IV (%)
**Fatigue**	73 (82,95)	54 (61.40)	19 (21,60)
**HFSR**	59 (67.05)	45 (51.11)	14 (15.91)
**Diarrhoea**	55 (62.50)	44 (50.00)	11 (12.50)
**Anorexia**	47 (53.41)	38 (43.18)	9 (10.23)
**Stomatitis**	33 (37.50)	27 (30.68)	6 (6.82)
**Anemia thrombocytopenia**	29 (33.00)	26 (29.55)	3 (3.41)
**Abdominal pain**	16 (18.18)	13 (14.77)	3 (3.41)
**Weight loss**	24 (27.27)	23 (26.14)	1 (1.13)
**Neutropenia**	4 (4.55)	3 (4.41)	1 (1.13)
**Nausea**	25 (28.41)	23 (26.14)	2 (2.27)
**Skin rash**	13 (14.77)	11 (12.50)	2 (2.27)
**Skin dryness**	3 (3.41)	3 (3.41)	0
**Hypertension**	13 (14.77)	12 (13.64)	1 (1.13)
**Voice alteration**	10 (11.36)	10 (11.36)	0
**General muscle weakness**	3 (3.41)	3 (3.41)	0

**Uncommon side effects**: gastrointestinal perforations (*N* = 2), acute pancreatitis (N = 1), digestive haemorrhages (*N* = 2), septic shock (*N* = 1), thromboembolic events (*N* = 2), and hiccups (*N* = 2)

Patients presented a median of 7 (Q1 = 4, Q3 = 9) different adverse reactions during therapy. All but one patient experienced at least one toxicity of any grade, of whom 61.4% with at least one grade III-IV. Grade III-IV side effects were mainly fatigue (21.6%), hand-foot skin reactions (HFSR) (15.9%), and diarrhoea (12.5%). No toxic death was observed. Toxicity led to dose modifications in 63.6% and therapy discontinuation in 5.7% of cases.

### Survival data and radiological response

The mOS and mPFS were 8.2 months (95% CI: 6.8–10.5) and 4.2 months (95% CI: 3.4–4.8) respectively. The OS rate at 6 months was 71% (65/92) (95% CI: 61%-79%), significantly higher than the 30% minimal efficiency level predefined in the statistical plan (*p*-value <0.001), meeting the clinical co-primary endpoint.

According to RECIST, partial response was observed in 7/92 patients (7.6%, 95%CI 2.2–13.0). In the 79 assessable patients, disease control at first evaluation (partial responses and stable diseases according to RECIST) was noted in 32/37 (80%) of the patients with consistent mR versus 24/42 (57%) in other patients (*p*-value 0.006) (Table 2).

**Table 2:** RECIST1.1 versus Metabolic Response classes in patients for whom both mR and RECIST assessment of response are available.

	Best RECIST response			
	PR	SD	PD	total
Early metabolic Response				
I	4	28	4	36
II	-	9	5	14
III	-	7	4	11
IV	-	8	8	16
Total	4	52	21	77

### Metabolic response analysis

MR data were available for 79 patients: 37 (46.8%) were classified as class I; 14 (17.7%) as class II; 11 (13.9%) as class III; and 17 (21.5%) as class IV. Within Class IV, 8 patients (10%) showed early metabolic disease progression.

**Fig 3:**
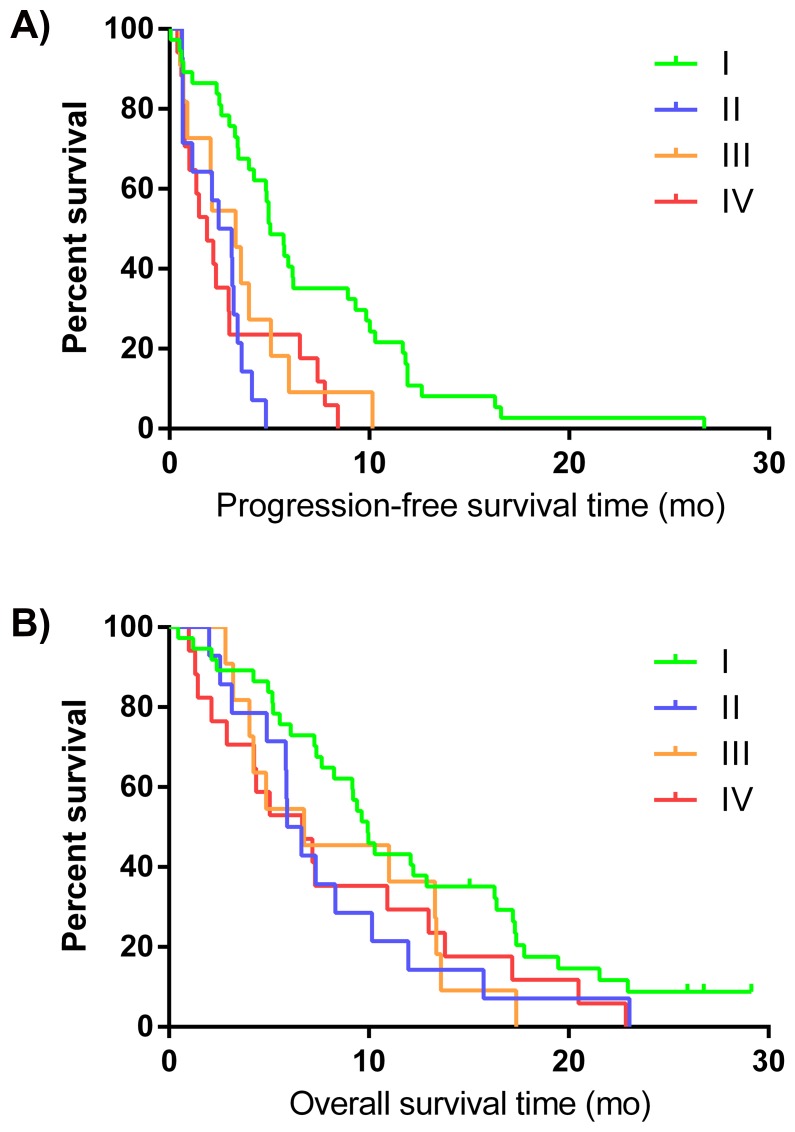
PFS* (A) and OS* (B) distribution according to the 4 classes of metabolic response. Class 1: no metabolic unresponsive lesion; Class 2: minority of unresponsive lesion among whole body target tumour load; Class 3: majority of whole body target tumour load does not respond; Class 4: all target lesions are non-responding, or, presence of progressive lesions [progression defined as >25% increase of FDG uptake on second PET, or appearance of a new lesion]. *from date of the second FDG PET-CT.

Patients without any metabolically non-responding lesions (Class I) performed better than patients with heterogeneous responses (Class II and III) or with a consistent non-response or progressive disease (Class IV). The difference between the four classes is statistically significant for mPFS (*p*-value <0.001) but not for mOS (*p*-value = 0.13). (Fig 3A and 3B)

**Fig 4:**
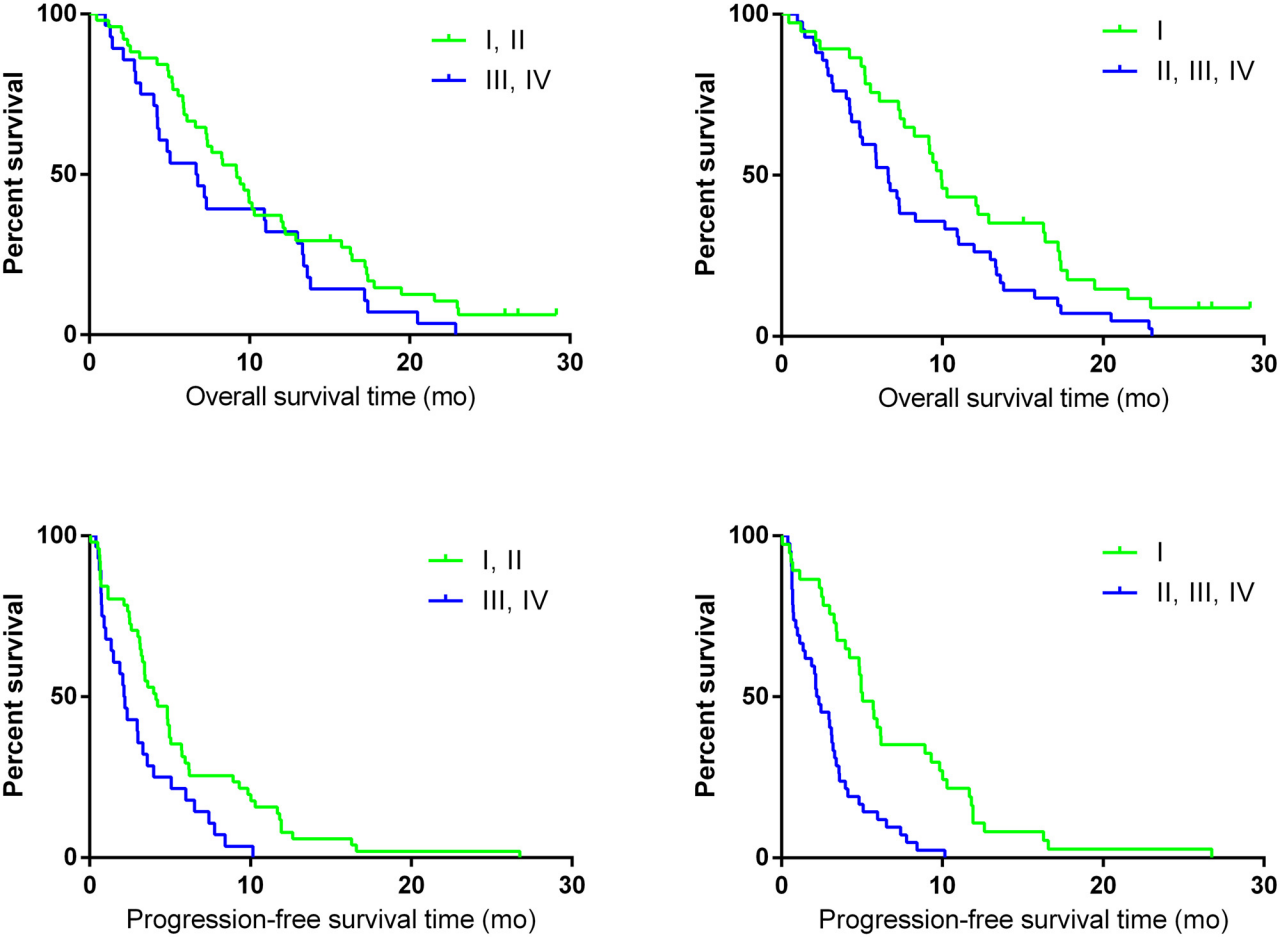
PFS and OS distribution according to the dichotomized mR classifications.

Two classifications were considered for reporting response in a dichotomized way according to mR heterogeneity among lesions: classes (I and II) versus classes (III and IV),[13] and classes (I) versus classes (II+III+IV). The first compares outcome according to the dominance of non-mR lesions within the tumor load, the second according to the consistence of mR (Table 3, Fig 4). “Using the “dominance” classification to define early metabolic non response, the second co-primary objective, which was to identify a prognostic value on survival for early metabolic assessment, was not met while it was successful to discriminate patients according to their outcome using the exploratory “consistence” classification.“Five of the 42 patients (12%) with at least one non-responding lesion remained free of disease progression at 6 months, versus 15 of the 37 class I patients (41%) (*p*-value 0.005).

**Table 3:** Correlation of mPFS and mOS with Dominance and Consistency of metabolic response.

mR classification	Inter-observer Kappa	Metabolic Classes of Response	mPFS	mOS
Dominance—Classes I+II versus classes III+IV	0.78	mR—(class I-II, N = 51)	4.1 months (95% CI, 3.1 to 5.0)	9.2 months (95% CI, 6.6 to 12.0)
		mNR—(class III-IV, N = 28)	2.2 months (95% CI, 1.0 to 3.3)	6.7 months (95% CI, 4.2 to 11.0)
		Hazard Ratio	0.52 (95%CI, 0.32 to 0.84) *p*-value 0.007	0.68 (95%CI, 0.42 to 1.09) *p*-value 0.10
Consistency—Class I versus classes II+III+IV	0.70	mR—(class I, N = 37)	5.0 months—(95%CI, 4.0 to 8.9)	9.9 months—(95%CI, 7.6 to 16.3)
		mNR—(class II-IV, N = 42)	2.3 months (95% CI, 1.3 to 3.1)	6.6 months (95% CI, 4.9 to 8.3)
		Hazard Ratio	0.34 (95%CI, 0.21 to 0.56) *p*-value <0.001	0.58(95%CI, 0.36 to 0.92) *p*-value 0.02

Multivariate analysis after stepwise variable selection of age, PS, number of previous chemotherapy lines, bevacizumab pretreatment, sex, Body Mass Index (BMI), HFSR occurrence and mR retained the absence of metabolically resistant lesion (class I) as the only variable significantly correlated with both mOS and mPFS (Table 4).

**Table 4:** Univariate and multivariate analysis for OS^¶^ and PFS^¶^.

	OS				PFS			
	UNIVARIATE		MULTIVARIATE		UNIVARIATE		MULTIVARIATE	
Variable	Hazard ratio (95% CI)	P-value	Hazard ratio (95% CI)	P-value	Hazard ratio (95% CI)	P-value	Hazard ratio (95% CI)	P-value
**mResponse Class I (vs II,III,IV)**	0.58 (0.36 to 0.92)	0.02	0.56 (0.35 to 0.89)	0.01	0.34 (0.21 to 0.56)	<0.001	0.29 (0.17 to 0.49)	<0.001
**ECOG PS 1 (vs 0)**	1.18 (0.74 to 1.88)	0.49			1.21 (0.77 to 1.92)	0.41		
**Number of previous chemotherapy lines (1 to 6)**	0.89 (0.73 to 1.10)	0.28			0.88 (0.69 to 1.11)	0.27		
**Previous treatment with bevacizumab (vs no)**	1.69 (1.05 to 2.72)	0.03	1.80 (1.10 to 2.92)	0.02	1.86 (1.16 to 2.98)	0.01	1.80 (1.12 to 2.91)	0.02
**KRAS mutation (vs WT)**	0.72 (0.45 to 1.13)	0.16			1.01 (0.65 to 1.58)	0.95		
**BMI ≥25 (vs <25)**	0.55 (0.34 to 0.87)	0.01	0.50 (0.31 to 0.81)	0.004	0.72 (0.46 to 1.14)	0.16		
**Age**	0.98 (0.96 to 1.01)	0.15			0.98 (0.96 to 1.01)	0.15		
**Male (vs female)**	0.68 (0.43 to 1.07)	0.09			0.62 (0.39 to 0.98)	0.04	0.53 (0.33 to 0.87)	0.01
**Occurrence HFSR before 2** ^**nd**^ **FDG PET-CT(vs no)**	0.54 (0.33to 0.89)	0.02	0.49 (0.30 to 0.81)	0.005	0.87 (0.55 to 1.38)	0.54		
**Occurrence HSFR** * **(vs no)**	0.59 (0.37 to 0.95)	0.03			0.81 (0.51 to 1.27)	0.35		

* Time dependent variable;

^¶^ from date second FDG PET-CT

## Discussion

Tumoral heterogeneity, described as the coexistence of genomically different subclones within a patient tumor load or to local environmental aspects, is recognized as a major determinant of resistance to treatment in solid tumors.[1–3] However, interlesional tumor heterogeneity in metastatic setting is not covered by current response assessment methods because of the analysis’ methodology performing averaging of responses among lesions. This prospective multicentric proof-of-concept study explored interlesional mR heterogeneity as a biomarker of treatment resistance in advanced solid tumors.

As previously reported in several solid tumors, FDG-PET/CT response assessment after one therapy cycle allows a rapid identification of non-responding lesions/patients, fulfilling the necessary conditions to become potentially a good predictive biomarker, which is crucial to avoid useless toxicity.[4,9–12,22,27] Moreover, significant progresses and implementation of standardized methodology for FDG-PET/CT imaging, including homogenization of imaging procedures and patient’s preparation, quality control and independent central analysis, now allows its use in multicentric trials.[24,27,28]

Studying tumoral heterogeneity requires assessing the response of the whole baseline metastatic tumor load without restriction in number nor site. However, existing morphological (WHO, RECIST) and metabolic (EORTC, PERCIST) response assessment methods do not take into account this response heterogeneity because they only consider a limited number of operator-selected target lesions and/or perform summing or averaging of response variables.[4,19,29,30] Moreover, being classically performed late during treatment, these assessment criteria measure response, while from a clinical point of view, it is the presence of non-response that triggers the need for treatment adaptation. For this, based on prior research, in order to optimize the negative predictive value (NPV) of mR assessment, a 15% cut-off value of SUVmax decrease instead of the standard 25–30% response cut-off value was chosen.[18,31] Such low cut-off value maximally avoids unjustified denial to a potentially active treatment regimen.

With regard to the characterization of response heterogeneity among lesions, this study adopted a multistep descriptive procedure. First, a lesion-by-lesion response analysis of all measurable lesions on baseline FDG-PET/CT without restriction of their number was performed applying the 15% cut-off for non-response. Then, a patient-based 4-class classification was applied, describing the presence and proportion of metabolically non-responding lesions among the whole-body tumor load.[13]

Using such methodology, 22% of the patients showed overall treatment resistance of whom 10% showed early metabolic disease progression at 3 weeks. This observation indicates the importance of performing the baseline FDG-PET/CT as close as possible before the start of the tested drug administration, because rapid disease progression during this timeframe could lead to false negative mR assessment.

On the other hand, after one treatment cycle, 32% of the patients showed heterogeneous metabolic responses combining resistant with potentially responding lesions (Class II and III). Of these, 18% showed non-mR in the minor, while 14% showed a non-mR in the major part of the tumor load. The proportion of heterogeneous response observed in this study is considerable, confirming earlier observation in an independent mCRC patient group treated with chemotherapy, where heterogeneity of mR was described in 67% of patients.[13] Other comparisons are impossible because information about heterogeneity is lacking in most available literature, which apply dichotomization to response assessment.[32–34]

Indeed, for clinical decision-making, the response assessment is generally reported dichotomously, because clinicians have to decide whether to continue or adapt the initiated treatment. Such information-reducing response reporting may only be adequate in case of homogeneous mR, but blurs useful information in case of response heterogeneity.

Outcome analysis in this study indicated that mPFS and mOS are comparable in patients bearing one or more metabolically resistant lesion. Only patients without any resistant lesion (class I) seemed to have a better outcome (mPFS and mOS) compared to all others. Therefore it seems that the presence but not the number/proportion of non-responding lesions is the most important prognostic determinant. Moreover, its value is reinforced by a multivariate analysis showing absence of any metabolically treatment resistant lesion as an independent prognostic factor for both PFS and OS.

A valid assessment of a predictive biomarker requires a significant level of activity of the regimen under study. This was achieved, as 71% of the included patients were still alive at 6 months, which was significantly higher than the minimal activity predefined in the study design. ITT analysis of the 92 eligible patients showed a mPFS of 4.2 months and a mOS of 8.2 months respectively, suggesting an overall beneficial effect for this drug combination compared to recent historical data with 2 months mPFS and 4–6 months mOS in the same clinical setting.[6,31,35–37]

Moreover, this study confirms the need for an effective predictive response biomarker for a sorafenib-containing regimen, because of the high toxicity profile together with the poor sensitivity of morphology-based imaging (CT/MRI) for detecting responses (only 8% of partial response according to RECIST) during treatment.[7,8,38]

A major application of standardized metabolic imaging is expected in early drug development (phase I-II) for two reasons: (i) as FDG-PET response analysis seems to be correlated with prognosis, it provides a rapid appraisal of the new drug activity even in small patient populations, and (ii) image-guided biopsies of resistant lesions could identify the molecular basis of treatment resistance by demonstrating genomic or epigenomic heterogeneity.

In this study for instance, half (47%) of the patients didn’t demonstrate any resistant lesion, indicating a remarkable activity level for such a heavily pre-treated patients population, unsuspected by classical morphological imaging.

Furthermore, in the metastatic setting, FDG-PET/CT may provide a tool for the identification of patients with one or very few metastatic sites resisting to treatment for whom the continuation of unchanged therapy carries a grim prognosis. This raises the potential of adding locoregional ablative treatments guided by the imaging of metabolic response, in order to achieve homogeneity of disease control and restore prognosis. If the current observation is confirmed by an ongoing multicentric trial, (clinicaltrials.gov NCT01929616), randomized prospective trials using early FDG-PET/CT response assessment as an interventional tool for targeting locoregional therapy (eg. surgery, radioembolization, radiofrequency ablation) will be justified.

Finally, in the absence of randomized data based on PET response, it remains to be proven whether the presence of metabolically non-responding lesions is a biomarker identifying more heterogeneous diseases with intrinsically a worse prognosis, or a genuine therapeutic predictive tool for a given treatment.

## Conclusions

Metabolic response assessment allows the early identification of treatment-resistant tumor sites. The presence of metabolically refractory lesions seems to negatively impact overall treatment outcome whatever their number, adding to the mounting evidence that tumour heterogeneity is a key element in cancer management.

Early assessment of mR heterogeneity is a potentially powerful predictive biomarker enabling the personalization of anticancer treatments by increasing their cost-effectiveness and sparing useless toxicities.

## Supporting Information

S1 Protocol Study protocol. (PDF)

S1 TREND Checklist TREND Checklist. (PDF)
