# Supplementary material for: The Prognostic Significance of Metabolic Response Heterogeneity in Metastatic Colorectal Cancer
Source: PLoS One. 2015 Sep 30;10(9):e0138341. doi: 10.1371/journal.pone.0138341 (PMC4589397; doi:10.1371/journal.pone.0138341)
Supplement: S1 Protocol — (PDF) [file pone.0138341.s001.pdf]

Sorafenib plus capecitabine efficacy assessment in patients with advanced pre-treated colorectal cancer

EudraCT number : 2010-023695-91

Protocol Version Date: October 29, 2010

Title: Sorafenib plus capecitabine efficacy assessment in patients with advanced pre-treated colorectal cancer.

Principal Investigator: Alain Hendlisz, MD - Institut Jules Bordet

Coordinating Center/Sponsor : Institut Jules Bordet

Co-Investigators:

Vassilis Golfinopoulos, MD, PhD; Research fellow, Institut Jules Bordet ULB

Amélie Deleporte, MD; Medicine department; Institut Jules Bordet ULB

Patrick Flamen, MD, PhD; Nuclear Medicine Department, Institut Jules Bordet ULB

Camilo Garcia, MD; Micolab, Central Core Lab for Molecular Imaging, Institut Jules Bordet ULB

Tatiana Besse-Hammer, MD; Medicine Department; Institut Jules Bordet ULB

Jean-Luc Van Laethem, MD, PhD, Hopital Universitaire Erasme, ULB

Thierry Delaunoit, MD; Entité Jolimontoise

Gauthier Demolin, MD; Hopital Saint Joseph, Liège

Ghislain Houbier, MD; Hopital Saint Joseph, Liège

Statistician: Marianne Paesmans - Institut Jules Bordet ULB

Data Center : Marianne Paesmans - Institut Jules Bordet ULB

Study Coordinator: Alain Hendlisz, MD - Institut Jules Bordet ULB

Agent(s): Sorafenib - Bayer Healthcare, Capecitabine - Roche

CONFIDENTIAL

This document is confidential. Do not disclose or use except as authorized.

SCHEMA

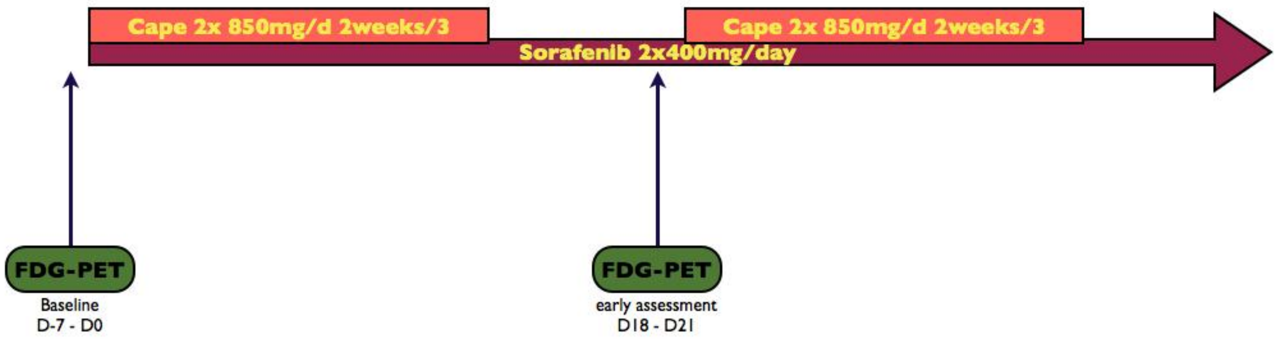
Cape 2x 850mg/d 2weeks/3
Cape 2x 850mg/d 2weeks/3
Sorafenib 2x400mg/day
FDG-PET
Baseline
D-7 - D0
FDG-PET
early assessment
D18 - D21

So More : Study Synopsis

**Title of Study**	**Sorafenib and Capecitabine in refractory Metastatic Colorectal Cancer : "So More" study**
**Indication**	Advanced chemorefractory colorectal cancer
**Treatment line**	**mCRC** 3d line mutated KRAS **or** 4th line wild-type KRAS
**Study Coordinator/ Principal investigator for IJB**	Alain Hendlisz MD, Unité d'Oncologie Digestive, Service de Médecine, Institut Jules Bordet, Université Libre de Bruxelles
**Primary endpoint**	a) To obtain a preliminary assessment about the activity of the combination by estimating overall survival of the study population at a fixed time point (6 months)<br><br>b) To compare as an exploratory analysis the overall survival of metabolic responders versus non-responders.
**Secondary endpoints**	• To estimate the progression-free survival distribution of the study population<br>• To determine the objective response rate of the study population as assessed by standard imaging.<br>• To describe the adverse reactions associated with the study regimen in the study population.<br>• To determine the correlation of early metabolic response, as assessed by FDG-PET/CT immediately before the first and the second cycles of treatment with the study regimen, with overall survival, progression-free survival, and response.<br>• To determine the correlation of *growth modulation index* (GMI), defined as the time to progression under the study regimen over the time to progression under the latest prior regimen administered to the patient, with overall survival and progression-free survival.
**Study design**	Prospective non-randomized phase II study

CONFIDENTIAL

This document is confidential. Do not disclose or use except as authorized.

	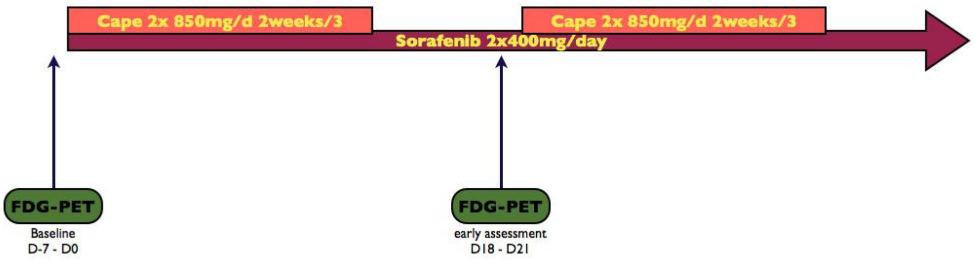
**Inclusion criteria**	• Participants must have histologically confirmed colorectal cancer that is metastatic or unresectable and for which standard curative or palliative measures do not exist or are no longer effective.<br>• All standard chemotherapy agents (fluoropyrimidines, irinotecan, and oxaliplatin) and monoclonal antibodies (bevacizumab, cetuximab, and panitumumab) are allowed as administered therapy before study entry. No more than two lines of treatment for metastatic or recurrent disease are allowed, except for patients with KRAS-wt tumors, for which third line with anti-EGFR agents is allowed.<br>• Age over 18 years.<br>• Life expectancy of greater than 12 weeks.<br>• ECOG performance status ≤ 1.<br>• Participants must have normal organ and marrow function as defined below:<br>• Leukocytes ≥ 3,000/mcL<br>• Absolute neutrophil count ≥ 1,500/mcL<br>• Platelets ≥ 100,000/mcL<br>• total bilirubin within 2 × normal institutional limits<br>• AST/ALT/PAKL levels ≤ 5 × institutional upper limit of normal<br>• creatinine within 2 × normal institutional limits or creatinine clearance ≥ 35mL/min<br>• Women of child-bearing potential and men must agree to use adequate contraception (hormonal or barrier method of birth control, abstinence) prior to study entry and for the duration of study participation. Should a woman become pregnant or suspect she is pregnant while participating in this study, she should inform her treating physician immediately.<br>• Ability to understand and the willingness to sign a written informed consent document.
**Exclusion criteria**	Participants who exhibit any of the following conditions at screening will not be eligible for admission into the study.<br><br>• Participants who have had chemotherapy or radiotherapy within 4 weeks prior to entering the study or those who have not recovered from adverse events

	due to agents administered more than 4 weeks earlier.<br>• Participants may not be receiving any other experimental agents.<br>• Participants with known brain metastases should be excluded from this clinical trial because of their poor prognosis and because they often develop progressive neurologic dysfunction that would confound the evaluation of neurologic and other adverse events.<br>• History of allergic reactions attributed to compounds of similar chemical or biologic composition to sorafenib or capecitabine.<br>• Bleeding diathesis, history of cardiovascular ischemic disease or cerebrovascular incident within the last six months, or major surgery within four weeks.<br>• Uncontrolled concurrent illness including, but not limited to ongoing or active infection, symptomatic congestive heart failure, unstable angina pectoris, cardiac arrhythmia, or psychiatric illness/social situations that would limit compliance with study requirements.<br>• Pregnant women are excluded from this study because sorafenib and capecitabine are antitumor agents with the potential for teratogenic or abortifacient effects. Because there is an unknown but potential risk of adverse events in nursing infants secondary to treatment of the mother with sorafenib or capecitabine, breastfeeding should be discontinued if the mother is treated with sorafenib or capecitabine. These potential risks may also apply to other agents used in this study.<br>• Uncontrolled Diabetes<br>• Individuals with a history of a different malignancy are ineligible except for the following circumstances. Individuals with a history of other malignancies are eligible if they have been disease-free for at least 5 years and are deemed by the investigator to be at low risk for recurrence of that malignancy. Individuals with the following cancers are eligible if diagnosed and treated within the past 5 years: cervical cancer *in situ*, and basal cell or squamous cell carcinoma of the skin.
**Eligibility criteria**	• Delay between assessment of screening criteria and first PET/CT < 21 days<br>• FDG PET/CT positive and metabolically assessable lesions (>2cm diameter on baseline diagnostic CT) and lesions with a SUVmax x 2 superior to the SUVmax in normal liver or blood pool in cardiac cavities (if liver abnormal) at the baseline FDG PET/CT.<br>• Blood glucose < 150 mg/dl at the time of FDG administration in diabetic patients. Insulin or oral anti-diabetic medication is not allowed on the days of PET/CT imaging.<br>• Blood glucose <120 mg/dl at the time of FDG administration in NON diabetic patients<br>• Respect of technical specifications to perform FDG PET/CT examinations from the Standard Procedures Imaging Manual (SPIM)<br>• Delay between the first PET/CT imaging and the start of Sorafenib-

Capecitabine < 7 days

- Second PET/CT imaging performed on D21 (range: D19-D23, with D1 as the first day of chemo administration)

Treatment doses

Agent	Dose	Route	Schedule	Cycle Length
Sorafenib	200mg in the morning, 400mg in the evening; escalation to 400mg twice daily after 1 cycle	Oral	Continuous dosing	21 days (3 weeks)
Capecitabine	850mg/m^2 twice daily	Oral	Days 1-14, weeks 1-2	

Sample size justification/statistical analysis

Sample size has been estimated in order to be able to test the null hypothesis that the overall survival rate at 6 months is less than 30%. This hypothesis will be tested using a binomial distribution. The study should be able to reject the null hypothesis, using a 1-sided test with $\alpha = 0.025$, with a power of 90% in case of a true overall survival ≥ 50% (rate at 6 months). The sample size required is 66 eligible patients (to be followed for 6 months minimum). Analysis will be done on all registered patients using an ITT approach on all eligible patients.

A co-primary endpoint is to compare the overall survival of patients assessed as early PET responders and of patients assessed as early PET non responders (the clinicians will remain blinded for PET response assessment). For this primary analysis, patients who will undergo the second PET assessment will be eligible and time zero for measuring survival will be the date of this second PET examination. It is anticipated that 95% of the patients will be eligible for the analysis with a 50% expected rate of early PET non-responders (result obtained from an unpublished study conducted at Jules-Bordet Institute). With 66 patients registered, we anticipate then that 63 patients will be available for the co-primary endpoint. With 63 patients and our assumption that the HR for the comparison between the survival distributions will be around 0.385 (based on the previously mentioned unpublished study), we will need using a two-sided logrank test at the 2.5% level (2.5% chosen because of the existence of 2 co-primary endpoints), 54 events (power of 90%). With 63 patients and a follow-up after accrual of 1 year, we should reach this number of 54 events. However, to account for another possible 5% drop-out (patient's refusal for undergoing the second PET examination for instance), sample size should be increased to 70 eligible patients.

CONFIDENTIAL

This document is confidential. Do not disclose or use except as authorized.

	The study is designed as a single-arm phase II study, with all patients accrued in one stage. No early stopping rules will be used.
**Number of sites**	4 Belgian sites (referring to PEPITA network PET centers in Belgium)
**Study duration**	2.5 years recruitment + 6 months follow-up = 3 years total

TABLE OF CONTENTS

1. Objectives 11

1.1 Study Design 11

1.2 Primary Objectives 11

1.3 Secondary Objectives 11

2. Background 12

2.1 Study Agent(s) 12

2.2 Study Disease 13

2.3 Rationale 14

2.4 Correlative Studies Background 17

3. Participant selection 19

3.1 Inclusion Criteria 19

3.2 Exclusion Criteria 20

3.3 Elibility Criteria 22

4. Registration procedures 22

4.1 General guidelines 22

4.2 Registration process 23

5. Treatment plan 23

5.1 Pre-treatment Criteria 24

5.2 Agent Administration 24

5.3 General Concomitant Medication and Supportive Care Guidelines 25

5.4 Duration of Therapy 25

5.5 Duration of Follow Up 25

5.6 Criteria for Removal from Study *25*

6. Expected toxicities and dosing delays/modifications 26

6.1 Anticipated Toxicities *26*

6.2 Dose Modifications/Delays *28*

7. Correlative/special studies 40

7.1 Early FDG-PET/CT *40*

7.2 Growth modulation index (GMI) *41*

8. Study calendar 41

9. Measurement of effect 42

9.1 Definitions *42*

9.2 Methods of evaluation of measurable disease *43*

9.3 FDG-PET(CT). *43*

9.4 Other Response Parameters *43*

10. Adverse event reporting requirements 44

10.1 Definitions *44*

10.2 Procedures for AE and SAE Recording *46*

10.3 Reporting Requirements *46*

10.4 Reporting to the Study Sponsor *47*

10.5 Reporting to the Institutional Review Board (IRB) *47*

10.6 Reporting to the principal Institutional Review Board (IRB) *48*

10.7 Reporting to competent authorities (CA) *48*

10.8 Reporting to Bayer *48*

10.9 Monitoring of Adverse Events and Period of Observation *48*

11. Data and safety monitoring 49

11.1 Data reporting 49

11.2 Monitoring 49

11.3 Annual Safety Reporting 50

12. Regulatory considerations 50

12.1 Protocol Review and Amendments 50

12.2 Informed Consent 50

12.3 Ethics and Good Clinical Practice (GCP) 50

12.4 Study Documentation 51

12.5 Records Retention 51

13. Statistical considerations 51

13.1 Sample Size/Accrual Rate 52

13.2 Stratification Factors 52

13.3 Analysis of Secondary Endpoints 52

13.4 Reporting and Exclusions 52

14. Investigator authorization procedure 53

15. Forms and procedures for collecting data 53

15.1 Case report forms and schedule for completion 53

15.2 Data flow 53

16. Quality assurance 54

16.1 Control of data consistency 54

16.2 Audits 54

17. Ethical considerations 55

17.1 Patient protection ... 55

17.2 Subject identification ... 55

17.3 Informed consent ... 55

18. Administrative responsibilities ... 56

18.1 The Study Coordinator ... 56

18.2 The Joint Study Management Team ... 56

19. Trial sponsorship and financing ... 56

20. Trial insurance ... 56

21. Publication plan ... 57

21.1 Publication and Presentation rules ... 57

22. References ... 59

23. Appendices ... 63

23.1 Performance status criteria ... 63

1. Objectives

1.1 Study Design

The study is designed as a single-arm phase II study to assess the overall survival of patients treated with the combination Sorafenib-Capecitabine.

As an exploratory analysis, the value of FDG-PET/CT as a predictive marker of overall survival in patients treated with the combination Sorafenib-Capecitabine will be tested. The hypothesis is that patients assessed as early PET non responders will have a worse survival compared to those assessed as early PET responders. Overall survival (OS) is the primary endpoint. Standard radiologic assessment will be done every six weeks. FDG-PET/CT will be done immediately before the first and the second cycles of treatment.

1.2 Primary Objectives

a) To obtain a preliminary assessment about the activity of the combination by estimating overall survival of the study population at a fixed time point (6 months)

b) To compare as an exploratory analysis the overall survival of metabolic responders versus non-responders.

1.3 Secondary Objectives

- To estimate the progression-free survival distribution of the study population
- To determine the objective response rate of the study population as assessed by standard imaging.
- To describe the adverse reactions associated with the study regimen in the study population.
- To determine the correlation of early metabolic response, as assessed by FDG-PET/CT immediately before the first and the second cycles of treatment with the study regimen, with overall survival, progression-free survival, and response rate.
- To determine the correlation of *growth modulation index* (GMI), defined as the time to progression under the study regimen over the time to progression under the latest prior regimen administered to the patient, with overall survival and progression-free survival.

2. Background

2.1 Study Agent(s)

2.1.1 Sorafenib

Sorafenib is a multikinase inhibitor that decreases tumor cell proliferation in vitro. Sorafenib inhibits tumor growth of a broad spectrum of human tumor xenografts in athymic mice accompanied by a reduction of tumor angiogenesis. Sorafenib inhibits the activity of targets present in the tumor cell (CRAF, BRAF, V600E BRAF, c-KIT, and FLT-3) and in the tumor vasculature (CRAF, VEGFR-2, VEGFR-3, and PDGFR-ß). RAF kinases are serine/threonine kinases, whereas c-KIT, FLT-3, VEGFR-2, VEGFR-3, and PDGFR-ß are receptor tyrosine kinases.

2.1.2 Capecitabine

Capecitabine is a non-cytotoxic fluoropyrimidine carbamate, which functions as an orally administered precursor of the cytotoxic moiety 5-fluorouracil (5-FU). Capecitabine is activated via several enzymatic steps. The enzyme involved in the final conversion to 5-FU, thymidine phosphorylase (ThyPase), is found in tumor tissues, but also in normal tissues, albeit usually at lower levels. There is evidence that the metabolism of 5-FU in the anabolic pathway blocks the methylation reaction of deoxyuridylic acid to thymidylic acid, thereby interfering with the synthesis of deoxyribonucleic acid (DNA). The incorporation of 5-FU also leads to inhibition of RNA and protein synthesis. Since DNA and RNA are essential for cell division and growth, the effect of 5-FU may be to create a thymidine deficiency that provokes unbalanced growth and death of a cell. The effects of DNA and RNA deprivation are most marked on those cells which proliferate more rapidly and which metabolize 5-FU at a more rapid rate.

2.1.3 Sorafenib plus capecitabine

In a recent double-blind phase II trial (Baselga 2009), Baselga et al. demonstrated a benefit associated with the addition of 400mg bid sorafenib to 1000mg/m^2 bid capecitabine in locally advanced or metastatic HER2-negative breast cancer who had received two or fewer chemotherapy regimens. The PFS in the experimental arm was 6.4 months versus 4.1 months in the capecitabine arm (HR: 0.58 [95% CI: 0.41-0.81], p=0.0006). However, significantly higher toxicities have been observed in the combined arm, especially hand-foot skin reaction (grade 3 or 4, 45% vs. 13%) although only 13.4% vs. 8% discontinued the treatment.

In a phase I study conducted at Jules-Bordet Institute, the sorafenib plus capecitabine combination was synergistic and feasible at the dose of 400mg bid for sorafenib and 850mg bid for capecitabine (Awada submitted).

2.2 Study Disease

2.2.1 Extent of the problem

With a 35/100.000/year incidence rate in the developed world, colorectal cancer affects about 150.000 people per year in Western Europe. Metastatic disease (metastatic colorectal cancer, mCRC) concerns about half of the patients, carrying a grim prognosis if unresectable with curative intent when diagnosed. Progresses in chemotherapy have been substantial over the last decade, allowing rare but well-advertised secondary resections of primarily unresectable metastatic disease. However, in the palliative setting, chemotherapy aims essentially at extending life expectancy and the use of all available drugs (fluoropyrimidines, oxaliplatin, irinotecan, bevacizumab, anti-EGFR antibodies) successively or concomitantly has increased patients' median overall survival to more than 20 months (Grothey 2005, Falcone 2007, Fuchs 2007, Cassidy 2007, Van Cutsem 2009). However, no drug or combination of drugs is able to achieve a cure for metastatic disease, and the tumor will eventually become resistant to all known medications, leading to the patient's death.

2.2.2 Current therapeutic strategies

Nowadays, most efforts in improving patients' outcome have been made in first and second line therapies. Combinations of all active cytotoxics (i.e. FOLFOXIRI regimen (Falcone 2007)) or with one (Fuchs 2007, Cassidy 2007, Van Cutsem 2009) or two biological agents (Saltz 2007, Meyerhardt 2007) to frontline irinotecan or oxaliplatin-based chemotherapy have been tested and some are still underway. For the clinicians, the high toxicity generally associated with those "super combinations" may seem out of proportion considering the palliative outcome, except if resection possibilities could be improved by a sufficient response to therapy. Most patients with advanced colorectal cancer will unfortunately never meet the requirements needed for a curative resection, due to disease extent and location or to poor general condition (elderly or frail patients) and it seems obvious that for them the treatment plan should favor disease control over tumor response.

Slowing tumor progression as a cancer management concept in selected patient populations gains momentum, as suggested by the results of several trials studying toxicity-sparing strategies (sequencing treatments, therapeutic pauses, maintenance treatments, etc). Those studies are reportedly associated with the same results in terms of disease control, progression-free survival or overall survival as classical approaches (treatment until progression or upfront treatment with the most effective combinations) (Koopman 2007, Mandalà 2009, Goldberg 2007, Maindrault-Goebel 2004).

CONFIDENTIAL

This document is confidential. Do not disclose or use except as authorized.

2.3 Rationale

2.3.1 The antiangiogenic strategy in mCRC

Studies with Bevacizumab (BV), a recombinant humanized monoclonal antibody targeting vascular endothelial growth factor (VEGF) have demonstrated that, in combination with chemotherapy, antiangiogenic treatment can significantly extend overall survival (OS), as well as improve response rate (RR) and progression free survival (PFS) (Fuchs 2007, Cassidy 2007, Hurwitz 2004, Giantonio 2007) in first and second line therapy of mCRC.

No data from randomized studies are available on the efficacy of antiangiogenic therapies in third line therapy, after failure of other potentially active drugs (irinotecan, oxaliplatin and anti-EGFR antibodies).

Bevacizumab activity seems applicable beyond classical tumor response evaluation. Data from a randomized study (Hurwitz 2004) suggest that non-responding patients receiving bevacizumab could have better overall and progression-free survival than non-responding patients without bevacizumab (Mass 2005).

More recently, a prospective observational cohort study (BRiTE) assessed continuation of bevacizumab beyond progression as an independent prognostic factor for better overall survival in multivariate analysis (Grothey 2007). Combination of additional complementary antiangiogenic agents or strategies could be able to overcome currently identified mechanisms of tumoral resistance to angiogenesis-targeted therapies (Cao 2009).

These results reinforce the notion that radiological response rate is neither the only nor the best way to evaluate the patients' benefit from a treatment (Louvet 2001, Tang 2007), especially when it comes to antiangiogenic or molecular targeted treatments.

There are few randomized studies about the best treatment for patients who successively have been treated and ultimately have developed resistance to all known chemotherapeutic agents in mCRC. The following table lists those trials. Available data suggest that no known treatment can improve actual response rates, but that some benefit in term of PFS or OS could still be achieved. Combination of BV with 5FU/LV (bolus or infusion) was associated in a recent non-randomized trial of 339 patients with a poor response rate (4%), failing to meet the primary endpoint. Nevertheless, remarkably, OS was recorded at 9 months. One must observe meanwhile that a phase II trial is not suitable to evaluate correctly overall survival, as results can be influenced by patient selection (Buyse 2000).

Table. "Last-line" trials in mCRC

Author	Combination	N	ORR (%)	TTP (mos)	OS (mos)
**Randomized studies**					
Derek 2007	Cetuximab	287	8	1.9	6.1
	vs.				
	BSC	285	0	1.8	4.6
Cunningham 2004	Cetuximab+CPT-11	218	22.9	4.1	8.6
	vs.				
	Cetuximab	111	10.8	1.5	6.9
Van Cutsem 2009	Panitumumab	231	10	2	8
	vs.				
	BSC	232	0	1.7	8
**Non-randomized studies**					
Scartozzi 2006	Cape-mmc	61	8	3	6
Matin 2005	Cape-trimethrexate	32	7.4	3.3	5.9
Chong 2005	Cape-mmc	36	15.2	5.4	9.3
Gubanski 2005	Cape	20	0	2.8	6.1
Lim 2005	Cape-mmc	21	4.8	2.6	6.8
Lièvre 2007	Beva+ folfox/folfiri	20	40	Na	Na
Emmanouilides 2004	Beva-5FU/FA	19	0	4	>6 (not reached)
Chen 2007	5FU/LV-beva	339	1	3.5	9
Zoran 2007	Cape+beva	28	14.3	3	14.3
Mc Collum 2006	Cape-thalidomide	34	0	2.6	7.1

2.3.2 New drug development and the use of Sorafenib in mCRC

Therapeutic options have grown fast recently in advanced colorectal cancer: improvements in surgery (liver, lung, peritoneal metastasis are no longer synonyms of incurable disease), locoregional approaches (intrahepatic artery chemotherapy, Yttrium^{90} radioembolization, etc.) and availability of new and efficient drugs have simultaneously improved and complicated advanced colorectal cancer management. This complexity will likely hinder the analysis of any new agent introduced in first or second line therapy and blur its impact on patient's survival. This is illustrated by the divergence in findings for cetuximab, associated with a proven survival benefit when administered in 3d or 4th line (Jonker 2007, Cunningham 2004), but not when administered in 1st line (Van Cutsem 2009). Treatment at the 3rd and 4th line of therapy appears consequently as the new frontier to overcome, and the setting to introduce a new drug if it comes to demonstrate quickly its impact on survival.

This document is confidential. Do not disclose or use except as authorized.

Sorafenib, which is an oral multi-kinase inhibitor targeting RAF kinase (a member of the RAF/RAS/MAPK pathway), vascular endothelial growth factor receptor (VEGFR), platelet-derived growth factor receptor (PDGFR), FMS-like tyrosine kinase 3 (FLT-3), c-kit and RET-receptor tyrosine kinase, has been recently approved for the treatment of renal and hepatocellular carcinoma. Only small phase I trials have included mCRC patients for treatment with this molecule, but showed encouraging results. The association of Sorafenib and Capecitabine is synergistic and feasible at the dose of 400mg Sorafenib bid and 850mg Capecitabine bid (Awada submitted).

In a recent double-blind phase II trial (Baselga 2009), Baselga et al. demonstrated a benefit associated with the addition of 400mg bid sorafenib to 1000mg/m^2 bid capecitabine in locally advanced or metastatic HER2-negative breast cancer who had received two or fewer chemotherapy regimens. The PFS in the experimental arm was 6.4 months versus 4.1 months in the capecitabine arm (HR: 0.58 [95% CI: 0.41-0.81], p=0.0006). However, significantly higher toxicities have been observed in the combined arm, especially hand-foot skin reaction (grade 3 or 4, 45% vs. 13%) although only 13.4% vs. 8% discontinued the treatment.

As suggested by early phase I data, and by the numerous ongoing studies with sorafenib in colorectal cancer (see following figure), this association deserves further interest in colorectal carcinoma. The mode of action of sorafenib remains unknown, even if it is widely associated with antiangiogenic and antiproliferative effects, mostly through RAF inhibition. Nevertheless, the significant toxicity of this association in an advanced, palliative setting will impose a quick definition of patients unlikely to draw any benefit from the treatment in order to spare them unnecessary side effects.

1	Recruiting	Study to Evaluate the Effects of Sorafenib if Combined With Chemotherapy (FOLFOX6 or FOLFIRI) in the Second-Line Treatment of Colorectal Cancer<br>Condition: Colorectal Neoplasms<br>Interventions: Drug: Sorafenib; Drug: Placebo; Drug: Oxaliplatin or Irinotecan; Drug: Leucovorin; Drug: 5-Fluorouracil
2	Recruiting	Phase I Trial of Sorafenib + FOLFIRI in Metastatic Colorectal Cancer<br>Condition: Metastatic Colorectal Cancer<br>Intervention: Drug: sorafenib + FOLFIRI
3	Recruiting	A Phase I Trial Evaluating mFOLFOX6 and Avastin With Nexavar as First-Line Treatment for Metastatic Colorectal Cancer<br>Condition: Metastatic Colorectal Cancer<br>Intervention: Drug: Sorafenib
4	Recruiting	Sorafenib and Cetuximab in Treating Patients With Metastatic Colorectal Cancer<br>Condition: Colorectal Cancer<br>Interventions: Biological: cetuximab; Drug: sorafenib tosylate; Genetic: proteomic profiling; Other: immunoenzyme technique; Other: immunohistochemistry staining method; Other: laboratory biomarker analysis; Other: pharmacological study; Procedure: biopsy
5	Recruiting	Sorafenib With Irinotecan in Metastatic Colorectal Cancer (mCRC) and K-RAS Mutation<br>Condition: Metastatic Colorectal Cancer<br>Intervention: Drug: Nexavar (Sorafenib) and irinotecan
6	Active, not recruiting	Sorafenib, Cetuximab, and Irinotecan in Treating Patients With Advanced or Metastatic Colorectal Cancer<br>Condition: Colorectal Cancer<br>Interventions: Biological: cetuximab; Drug: irinotecan hydrochloride; Drug: sorafenib tosylate
7	Suspended	Sorafenib and Bevacizumab in Treating Patients With Metastatic Colorectal Cancer<br>Condition: Colorectal Cancer<br>Interventions: Biological: bevacizumab; Drug: sorafenib tosylate; Other: laboratory biomarker analysis; Other: pharmacological study
8	Recruiting	Study of Modified FOLFOX6 Plus or Minus Sorafenib in Stage IV Metastatic Colorectal Carcinoma (mCRC) Subjects<br>Conditions: Metastatic; Colorectal Cancer<br>Interventions: Drug: Sorafenib (Nexavar, BAY43-9006) + mFOLFOX6 (5-FU, levo-leucovorin, oxaliplatin); Drug: Matching placebo + mFOLFOX6 (5-FU, levo-leucovorin, oxaliplatin)
9	Recruiting	Sorafenib and FOLFIRI Regimen in 2nd Colorectal Cancer (CRC) After Failure of Oxaliplatin Treatment<br>Condition: Colorectal Neoplasms<br>Interventions: Drug: sorafenib; Drug: FOLFIRI
10	Recruiting	Study on the Influence of Sunitinib and Sorafenib on Fatigue, Quality of Life and Depression in Patients With Metastatic Renal Cell Cancer or Gastrointestinal Stromal Tumor (GIST)<br>Conditions: Renal Cell Cancer; Colorectal Cancer; GIST<br>Intervention: Other: Questionnaires
11	Recruiting	Sorafenib, Pemetrexed, and Cisplatin in Treating Patients With Advanced Solid Tumors<br>Conditions: Breast Cancer; Colorectal Cancer; Head and Neck Cancer; Lung Cancer; Malignant Mesothelioma; Pancreatic Cancer; Prostate Cancer; Sarcoma<br>Interventions: Drug: cisplatin; Drug: pemetrexed disodium; Drug: sorafenib tosylate
12	Recruiting	External-Beam Radiation Therapy, Capecitabine, and Sorafenib in Treating Patients With Locally Advanced Rectal Cancer<br>Condition: Colorectal Cancer<br>Interventions: Drug: capecitabine; Drug: sorafenib tosylate; Procedure: neoadjuvant therapy; Procedure: therapeutic conventional surgery; Radiation: radiation therapy

2.4 Correlative Studies Background

2.4.1 Early FDG-PET/CT

Standard radiological response measurements (RECIST criteria, modified RECIST, WHO) rely entirely upon measuring the size of the tumor with CT, ultrasound, or MRI, and are only applicable under restrictive conditions (well defined lesions, adequate minimum size, at least six weeks of chemotherapy). Response rates in advanced solid tumors are poorly correlated with other patients' outcomes, such as PFS and OS (Buyse 2000, Johnson 2006).

Several early response detection techniques are potentially emerging: serial FDG-PET-CT, dynamic MRI (DCE-MRI) and diffusion MR techniques, and Circulating Tumor Cells (CTCs). Among these, FDG-PET-CT is the most studied and promising. It is widely available in Belgium. Its value in detecting early metabolic changes predictive of later outcome is currently widely assessed (Byström 2009, Hendlisz 2009).

Recent data suggest that serial FDG-PET tumoral metabolic assessment is a reliable tool for early detection of refractory disease. A Belgian group prospectively included 42 mCRC patients undergoing first or second line chemotherapy. A serial FDG-PET was performed at baseline and 15 days after the first cycle of chemotherapy. The metabolic changes were compared to the morphologic response evaluated on CT by RECIST criteria. At interim analysis, 28 patients were available for comparative metabolic and morphological analysis of 88 lesions. A RECIST response was observed in 6/14 (43%) PET-responding patients and in 0/14

(0%) PET non-responding patients (p=0.02). This suggests that FDG-PET may be used to early detect the non-responders.

A FDG-PET driven metabolic study with similar design has been conducted in 50 advanced head and neck cancer patients treated with the association of sorafenib and capecitabine. The results of this study are still awaited and will probably be very useful to define a predictive tool to assess capecitabine-sorafenib combination outcome.

2.4.2 Growth modulation index (GMI)

Von Hoff has proposed a design that could be more efficient: to use each patient as his own control, by calculating the ratio of TTP with the treatment under investigation (TTP2) over the TTP with the last treatment the patient received (TTP1) (Von Hoff 1998) and using this ratio as primary endpoint. He proposed a ratio of 1.33 as the cut-off value over which a treatment should be considered active (i.e. observation of "response" for an individual patient), assuming a baseline ratio (i.e. no treatment effect) of at most 1.00. This proposal has clear biological rationale: reversal of the usually observed trend towards shorter TTPs in successive treatments should demonstrate change in the cancer's natural history, and, therefore, proof of value for the experimental drug.

To explore this proposal, Mick et al (Mick 2000) designed a simulation of clinical trials with different number of patients and expected results, additionally proposing the alternative value of 1.0 as cut-off for success and 0.7 as baseline. They found that a phase II study with 70 patients would have 84% power to detect a hazard ratio (HR) of 1.5, assuming correlation 0.5 between TTP1 and TTP2, a trial with two years of accrual and two years of follow-up, and using Von Hoff's suggested values for clinical efficacy. Fewer patients would be needed for larger HRs and more patients for smaller expected HRs. However, there are few data about expected correlations between TTP1 and TTP2 in specific clinical settings which might be a practical problem as this correlation has a large impact on sample size.

This strategy has been tested in prospective trials. Bonetti et al reported a phase II trial of oxaliplatin plus 5-fluorouracil plus leucovorin as second-line treatment for colorectal cancer (CRC) patients (Bonetti 2001). The TTP2/TTP1 ratio (referred to as *growth modulation index*, GMI) was 1.33 or larger in 47% (16/34) of the patients. Correlation between TTP1 and TTP2 was significant ($r = 0.514$, $p < 0.002$). Median TTP1 was 13 weeks and median TTP2 was 31 weeks ($p = 0.0081$). Comella et al presented a phase II trial of oxaliplatin plus raltitrexed plus 5-fluorouracil plus leucovorin in pre-treated CRC patients (Comella 2002). The TTP2/TTP1 ratio was 1.33 or larger (range 0.2-2.5) in 40% (16/40) of the patients who had progressed, and seemed unrelated with previous chemosensitivity. In both these trials, a ratio of 1.33 or more in a large (but not specified so far) percentage of patients could predict the favorable result of the trials.

3. Participant selection

Laboratory tests required for eligibility must be completed within 14 days prior to study entry. Baseline radiologic measurements for documentation of measurable disease must be documented from tests within 14 days of study entry. Other non-laboratory tests must be performed within 30 days of study entry.

3.1 Inclusion Criteria

Participants must meet the following criteria on screening examination to be eligible to participate in the study:

3.1.1 Participants must have histologically confirmed colorectal cancer that is metastatic or unresectable and for which standard curative or palliative measures do not exist or are no longer effective.

3.1.2 All standard chemotherapy agents (fluoropyrimidines, irinotecan, and oxaliplatin) and monoclonal antibodies (bevacizumab, cetuximab, and panitumumab) are allowed as administered therapy before study entry. No more than two lines of treatment for metastatic or recurrent disease are allowed, except for patients with KRAS-wt tumors, for which third line with anti-EGFR agents is allowed.

3.1.3 Age 18 years or more.

3.1.4 Life expectancy of greater than 12 weeks.

3.1.5 ECOG performance status ≤ 1.

3.1.6 Participants must have normal organ and marrow function as defined below:

- Leukocytes ≥ 3,000/mcL
- Absolute neutrophil count ≥ 1,500/mcL
- Platelets ≥ 100,000/mcL
- total bilirubin within 2 × normal institutional limits
- AST/ALT/PAKL levels ≤ 5 × institutional upper limit of normal

- creatinine within 2 × normal institutional limits or creatinine clearance $\geq$ 35mL/min

3.1.7 Women of child-bearing potential and men must agree to use adequate contraception (hormonal or barrier method of birth control, abstinence) prior to study entry and for the duration of study participation. Should a woman become pregnant or suspect she is pregnant while participating in this study, she should inform her treating physician immediately.

3.1.8 Ability to understand and the willingness to sign a written informed consent document.

3.2 Exclusion Criteria

Participants who exhibit any of the following conditions at screening will not be eligible for admission into the study.

3.2.1 Participants who have had chemotherapy or radiotherapy within 4 weeks prior to entering the study or those who have not recovered from adverse events due to agents administered more than 4 weeks earlier.

3.2.2 Participants may not be receiving any other study agents.

3.2.3 Participants with known brain metastases should be excluded from this clinical trial because of their poor prognosis and because they often develop progressive neurologic dysfunction that would confound the evaluation of neurologic and other adverse events.

3.2.4 History of allergic reactions attributed to compounds of similar chemical or biologic composition to sorafenib or capecitabine.

3.2.5 Bleeding diathesis, history of cardiovascular ischemic disease or cerebrovascular incident within the last six months, or major surgery within four weeks.

3.2.6 Uncontrolled concurrent illness including, but not limited to ongoing or active infection, symptomatic congestive heart failure, unstable angina pectoris, cardiac arrhythmia, or psychiatric illness/social situations that would limit compliance with study requirements.

3.2.7 Uncontrolled Diabetes

3.2.8 Pregnant women are excluded from this study because sorafenib and capecitabine are antitumor agents with the potential for teratogenic or abortifacient ef-

fects. Because there is an unknown but potential risk of adverse events in nursing infants secondary to treatment of the mother with sorafenib or capecitabine, breastfeeding should be discontinued if the mother is treated with sorafenib or capecitabine. These potential risks may also apply to other agents used in this study.

3.2.9 Individuals with a history of a different malignancy are ineligible except for the following circumstances. Individuals with a history of other malignancies are eligible if they have been disease-free for at least 5 years and are deemed by the investigator to be at low risk for recurrence of that malignancy. Individuals with the following cancers are eligible if diagnosed and treated within the past 5 years: cervical cancer *in situ*, and basal cell or squamous cell carcinoma of the skin.

3.3 Eligibility Criteria

3.3.1 Delay between assessment of screening criteria and first PET/CT < 21 days

3.3.2 FDG PET/CT positive and metabolically assessable lesions (>2cm diameter on baseline diagnostic CT) and lesions with a SUVmax x 2 superior to the SU-Vmax in normal liver or blood pool in cardiac cavities (if liver abnormal) at the baseline FDG PET/CT.

3.3.3 Blood glucose < 150 mg/dl at the time of FDG administration in diabetic patients. Insulin or oral anti-diabetic medication is not allowed on the days of PET/CT imaging.

3.3.4 Blood glucose <120 mg/dl at the time of FDG administration in NON diabetic patients

3.3.5 Respect of technical specifications to perform FDG PET/CT examinations from the Standard Procedures Imaging Manual (SPIM)

3.3.6 Delay between the first PET/CT imaging and the start of Sorafenib-Capecitabine < 7 days

3.3.7 Second PET/CT imaging performed on D21 (range: D19-D23, with D1 as the first day of chemo administration)

4. Registration procedures

4.1 General guidelines

Institutions will register eligible participants with the Data Center of Jules-Bordet Institute(by fax or by mail). Registration must occur prior to the initiation of therapy. Any participant not registered to the protocol before treatment begins will be considered ineligible and registration will be denied.

A member of the study team will confirm eligibility criteria and complete the protocol-specific eligibility checklist.

Following registration, participants may begin protocol treatment. Issues that would cause treatment delays should be discussed with the Principal Investigator. If a participant does not receive protocol therapy following registration, the participant’s protocol status must be changed. Notify the Data Center of participant status changes as soon as possible.

CONFIDENTIAL

This document is confidential. Do not disclose or use except as authorized.

4.2 Registration process

The Data Center staff is accessible on Monday through Friday, from 8:30 am to 16:30 pm.

The registration procedures are as follows:

1. Obtain written informed consent from the participant prior to the performance of any study-related procedures or assessments.

2. Complete the protocol-specific eligibility checklist using the eligibility assessment documented in the participant's medical/research record. **To be eligible for registration to the study, the participant must meet each inclusion and exclusion criteria listed on the eligibility checklist**.

3. Fax or mail the eligibility checklist(s) and all pages of the consent form(s) to the Data Center at +32 2 541 33 97.

4. The Data Center will validate eligibility and register the participant on the study.

5. The Data Center will send confirmation of the registration to the person initiating the registration immediately following the registration (the delay will not exceed one workday).

5. Treatment plan

Treatment will be administered on an outpatient basis. Expected toxicities and potential risks as well as dose modifications for sorafenib and capecitabine are described previously. No investigational or commercial agents or therapies other than those described below may be administered with the intent to treat the participant's malignancy.

**Agent**	**Dose**	**Route**	**Schedule**	**Cycle Length**
Sorafenib	200mg in the morning,<br>400mg in the evening;<br>escalation to 400mg twice daily	Oral	Continuous dosing	21 days (3 weeks)
Capecitabine	850mg/m^2 twice daily	Oral	Days 1-14, weeks 1-2	

5.1 Pre-treatment Criteria

5.1.1 Cycle 1, Day 1: See inclusion and exclusion criteria.

5.1.2 Subsequent Cycles: See dose reduction guidelines.

5.2 Agent Administration

5.2.1 Sorafenib

The study dose of sorafenib is 600mg (one tablet of 200mg in the morning and two tablets of 200mg in the evening) with escalation to 400mg (two tablets of 200mg) twice daily (equivalent to a total daily dose of 800mg). It is recommended that sorafenib should be administered without food or with a low or moderate fat meal. If the patient intends to have a high-fat meal, sorafenib tablets should be taken at least 1 hour before or 2 hours after the meal. The tablets should be swallowed with a glass of water.

5.2.2 Capecitabine

The study dose of capecitabine is 850mg/m^2 twice daily for 14 days, followed by a 7-day rest period. Capecitabine tablets should be swallowed with water within 30 minutes after a meal. Treatment should be discontinued if progressive disease or intolerable toxicity is observed. Toxicity due to Capecitabine administration may be managed by symptomatic treatment or modification of the dose (treatment interruption or dose reduction). Once the dose has been reduced, it should not be increased at a later time. For those toxicities considered by the treating physician to be unlikely to become serious or life-threatening, e.g. alopecia, altered taste, nail changes, treatment can be continued at the same dose without reduction or interruption. Patients taking Capecitabine should be informed of the need to interrupt treatment immediately if moderate or severe toxicity occurs. Doses of Capecitabine omitted for toxicity are not replaced. The recommended dose modifications for toxicity will be presented in table b .

5.2.3 Early FDG-PET/CT

Increased glycolysis is one of the hallmarks of cancer. FDG, an analogue of glucose labeled with a positron emitting isotope of Fluor (F18) is actively taken up in cancer cells of many tumor types. The positrons emitted by the FDG are detected by a dedicated camera, enabling the visualization of cellular glycolytic activity (Gambhir 2002).

There seems to be a consensus that two weeks is an appropriate time to detect change in metabolic activity and therefore to assess a patient under treatment using FDG-PET. The technique has already been used to prospectively define the therapeutic strategy in adenocarcinoma of the esophagogastric junction, with major histopathologic remissions being observed exclusively in metabolic responders (Lordick 2007).

Serial FDG PET/CT consists in performing a scan at baseline and shortly after the administration of the drug. The two PET/CT need to be performed in strictly identical and standardized conditions, both physiologically as technically. One most critical parameter is the timing between the tracer administration and the start of the imaging which should not differ by more than 10 minutes between the two PET scans.

5.3 General Concomitant Medication and Supportive Care Guidelines

Concomitant medications and supportive care measures (e.g. antiemetic or pain medications) should be administered according to investigator discretion, with appropriate care for potential drug interactions.

5.4 Duration of Therapy

Duration of therapy will depend on individual response, evidence of disease progression and tolerance. In the absence of treatment delays due to adverse events, treatment may continue for 6 cycles or until one of the following criteria applies:

- Disease progression,
- Concurrent illness that prevents further administration of treatment,
- Unacceptable adverse event(s),
- Participant decides to withdraw from the study, or
- General or specific changes in the participant's condition render the participant unacceptable for further treatment in the opinion of the treating investigator.

5.5 Duration of Follow Up

Participants will be followed for one year after removal from study or until death, whichever occurs first.

5.6 Criteria for Removal from Study

Study treatment will be stopped when progression of disease or unacceptable toxicities occur. The follow-up of the patients will go on. The reason for stopping study treatment and the date of stop must be documented in the study-specific case report form (CRF). Alternative care options will be discussed with the participant.

In the event of unusual or life-threatening complications, participating investigators must immediately notify the Principal Investigator, Dr Alain Hendlisz, MD, at tel. +32 2 541 31 96.

6. Expected toxicities and dosing delays/modifications

Dose delays and modifications will be made using the following recommendations. Toxicity assessments will be done using the CTEP active version of the NCI Common Terminology Criteria for Adverse Events (CTCAE), which is located on the CTEP website at: http://ctep.cancer.gov/protocoldevelopment/electronic_applications/docs/ctcaev3.pdf.

In the case of toxicity, appropriate medical treatment should be used (including anti-emetics, anti-diarrheals, etc.).

All adverse events experienced by participants will be collected from the time of the first dose of study treatment, through the study and until the final study visit. Participants continuing to experience toxicity at the off study visit may be contacted for additional assessments until the toxicity has resolved or is deemed irreversible.

6.1 Anticipated Toxicities

A list of the adverse events and potential risks associated with the agents administered in this study appear below and will determine whether dose delays and modifications will be made or whether the event requires expedited reporting *in addition* to routine reporting.

6.1.1 Adverse Events for sorafenib

The most common adverse events for sorafenib are listed below. For more details the reader is referred to the package insert of the drug.

Dermatological toxicities: Hand-foot skin reaction (palmar-plantar erythrodysesthesia) and rash represent the most common adverse drug reactions with Sorafenib. Rash and hand-foot skin reaction are usually CTC (Common Toxicity Criteria) Grade 1 and 2 and generally appear during the first six weeks of treatment with Sorafenib. Management of dermatological toxicities may include topical therapies for symptomatic relief, temporary treatment interruption or dose modification of Sorafenib, or in severe or persistent cases, permanent discontinuation of Sorafenib.

Hypertension: An increased incidence of arterial hypertension was observed in Sorafenib-treated patients. Hypertension was usually mild to moderate, occurred early in the course of treatment, and was amenable to management with standard antihypertensive therapy. Blood pressure should be monitored regularly and treated, if required, in accordance with standard medical practice. In cases of severe or persistent hypertension, or hypertensive crisis despite institution of antihypertensive therapy, permanent discontinuation of Sorafenib should be considered.

CONFIDENTIAL

This document is confidential. Do not disclose or use except as authorized.

Hemorrhage: An increased risk of bleeding may occur following Sorafenib administration. If any bleeding event necessitates medical intervention it is recommended that permanent discontinuation of Sorafenib should be considered.

Cardiac ischemia or infarction: In a randomized, placebo-controlled, double-blind study the incidence of treatment-emergent cardiac ischemia/infarction events was higher in the Sorafenib group (2.9%) compared with the placebo group (0.4%). In another study, the incidence of treatment-emergent cardiac ischemia/infarction events was 2.7% in Sorafenib patients compared with 1.3% in the placebo group. Patients with unstable coronary artery disease or recent myocardial infarction were excluded from these studies. Temporary or permanent discontinuation of Sorafenib should be considered in patients who develop cardiac ischemia or infarction.

Gastrointestinal perforation: Gastrointestinal perforation is an uncommon event and has been reported in less than 1% of patients taking sorafenib. In some cases this was not associated with apparent intra-abdominal tumor. Sorafenib therapy should be discontinued.

6.1.2 Adverse Events for capecitabine

The most common adverse events for capecitabine are listed below. For more information the reader is referred to the package insert of the drug.

Dose limiting toxicities include diarrhea, abdominal pain, nausea, stomatitis and hand-foot syndrome (hand-foot skin reaction, palmar-plantar erythrodysesthesia). Most adverse reactions are reversible and do not require permanent discontinuation of therapy, although doses may need to be withheld or reduced.

Diarrhea. Patients with severe diarrhea should be carefully monitored and given fluid and electrolyte replacement if they become dehydrated. Standard antidiarrheal treatments (e.g. loperamide) may be used. NCIC CTC grade 2 diarrhea is defined as an increase of 4 to 6 stools/day or nocturnal stools, grade 3 diarrhea as an increase of 7 to 9 stools/day or incontinence and malabsorption. Grade 4 diarrhea is an increase of ≥10 stools/day or grossly bloody diarrhea or the need for parenteral support. Dose reduction should be applied as necessary.

Dehydration. Dehydration should be prevented or corrected at the onset. Patients with anorexia, asthenia, nausea, vomiting or diarrhea may rapidly become dehydrated. If Grade 2 (or higher) dehydration occurs, Capecitabine treatment should be immediately interrupted and the dehydration corrected. Treatment should not be restarted until the patient is rehydrated and any precipitating causes have been corrected or controlled. Dose modifications applied should be applied for the precipitating adverse event as necessary.

Hand-foot syndrome (also known as hand-foot skin reaction or palmar-plantar erythrodysesthesia or chemotherapy induced acral erythema). Grade 1 hand- foot syndrome is defined as

numbness, dysesthesia/paresthesia, tingling, painless swelling or erythema of the hands or feet or discomfort which does not disrupt the patient's normal activities. Grade 2 hand- foot syndrome is painful erythema and swelling of the hands or feet or discomfort affecting the patient's activities of daily living. Grade 3 hand- foot syndrome is moist desquamation, ulceration, blistering and severe pain of the hands or feet or severe discomfort that causes the patient to be unable to work or perform activities of daily living. If grade 2 or 3 hand- foot syndrome occurs, administration of Capecitabine should be interrupted until the event resolves or decreases in intensity to grade 1. Following grade 3 hand-foot syndrome, subsequent doses of Capecitabine should be decreased.

Cardiotoxicity. Cardiotoxicity has been associated with fluoropyrimidine therapy, including myocardial infarction, angina, dysrhythmias, cardiogenic shock, sudden death and electrocardiographic changes. These adverse reactions may be more common in patients with a prior history of coronary artery disease. Cardiac arrhythmias, angina pectoris, myocardial infarction, heart failure and cardiomyopathy have been reported in patients receiving Capecitabine. Caution must be exercised in patients with history of significant cardiac disease, arrhythmias and angina pectoris.

6.2 Dose Modifications/Delays

All toxicities should be graded according to the active version of NCI-CTCAE. Doses of capecitabine or sorafenib/placebo may be reduced/interrupted in the setting of any AE that is:

- Not controlled by optimal supportive care, or
- Not tolerated due to symptomatology, disfigurement, or interference with normal daily activities, regardless of severity.

Dose modifications will be based on the worst grade of an AE or laboratory abnormality during a given cycle. If multiple AEs are observed, the dose modification should be based on the most severe (ie, worst grade) event.

Treatment delays of sorafenib/placebo for up to 21 days are acceptable in order to allow for resolution of symptoms to NCI-CTCAE v4.0 Grade 1 or less. For subjects with NCI-CTCAE v4.0 Grade 2 or greater toxicities at baseline (screening), resolution of symptoms to baseline severity (ie, Grade 2 or greater) is acceptable.

Subjects requiring interruption of study treatment for more than 21 days will be discontinued from study treatment. These subjects will, however, be followed until death or overall completion of the trial, whichever comes first.

If possible, symptoms should be managed symptomatically. In the case of toxicity, appropriate medical treatment should be used (including anti-emetics, anti-diarrheal, etc.).

CONFIDENTIAL

This document is confidential. Do not disclose or use except as authorized.

Management of suspected adverse drug reactions may require temporary interruption or dose reduction of sorafenib therapy. When dose reduction is necessary, the sorafenib dose should be reduced to two tablets of 200mg once daily.

Toxicity due to capecitabine administration may be managed by symptomatic treatment or modification of the dose (treatment interruption or dose reduction). Once the dose has been reduced, it should not be increased at a later time. For those toxicities considered by the treating physician to be unlikely to become serious or life-threatening, e.g. alopecia, altered taste, nail changes, treatment can be continued at the same dose without reduction or interruption. Patients taking capecitabine should be informed of the need to interrupt treatment immediately according to the following template. Doses of capecitabine omitted for toxicity are not replaced. The recommended dose modifications for toxicity are presented in the following tables.

Table a. Pre-defined Dose levels for Sorafenib and Capecitabine

	0	-1	-2	-3
Sorafenib				
Cycle 1*	200mg in the morning, 400mg in the evening	200mg bid	200mg bid, every other day	-
Subsequent cycles	400mg bid	200mg in the morning, 400mg in the evening	200mg bid	200mg bid, every other day
Capecitabine				
All cycles	850mg/sqm bid	637.5mg/sqm bid	425mg/sqm bid	-

*Applicable to subsequent cycles if the sorafenib dose is not escalated to 400mg bid.

6.2.1.1 Dose modification for hematologic toxicities

In general, doses of sorafenib/placebo should not be reduced for hematologic events, except for Grade-4 hematologic toxicities. Any subject experiencing any of the following hematologic toxicities should have capecitabine therapy held until the toxicity has resolved to Grade 1 or less.

- Absolute neutrophil count < 1,000/mm^3 (≥ Grade 3) and/or febrile neutropenia for > 7 days.

- Hemoglobin < 8 g/dL (≥ Grade 3).
- Platelet count ≤ 50,000/mm^3 (≥ Grade 3).

At the re-start of capecitabine, the dose of capecitabine should be reduced one level. **Once a dose reduction occurs the dose cannot be re-escalated**. Additional dose reductions may occur as needed in subsequent cycles. If a subject requires dose reduction below capecitabine 500 mg/m^2 twice daily or sorafenib/placebo 200 mg/1 placebo tablet twice daily, every other day, study drug must be permanently discontinued.

If the described hematologic toxicity persists for more than 21days, the subject should discontinue capecitabine treatment and, therefore, also sorafenib/placebo.

Dose modifications of capecitabine and sorafenib for hematologic toxicities are outlined in Table b.

Table b: Dose Modifications for Hematologic Toxicities

Toxicity	ANC/AGC ($x 10^9/L$)	Hemoglobin (g/dL)	Platelets ($x 10^9/L$)	Capecitabine	Sorafenib/ Placebo
**Grade 1**	≥ 1.5	< LLN – 10.0	≥75	No change	No change
**Grade 2**	≥ 1.0 to < 1.5	< 10.0 – 8.0	≥ 50 to < 75	Reduce by one dose level	No change
**Grade 3**	≥ 0.5 to < 1.0	< 8.0 – 6.5	≥ 25 to < 50	Delay drug until toxicity has resolved to Grade 2 or less, then reduce by one dose level	No change
**Grade 4**	< 0.5	Life-threatening consequence; urgent intervention indicated	< 25	Delay drug until toxicity has resolved to Grade 2 or less, then reduce by two dose levels	Delay drug until toxicity has resolved to Grade 2 or less, then reduce by one dose level
**Febrile Neutropenia**	—	—	—	Delay drug until toxicity has resolved to Grade 2 or less, then reduce by two dose levels	No change[a]

ANC=Absolute neutrophil count ; AGC=absolute granulocyte count

a: Subjects who experience febrile neutropenia associated with grade-4 neutropenia should have sorafenib/placebo held until toxicity has resolved to Grade 2 or less; when sorafenib/placebo is restarted, reduce by one dose level.

Granulocyte colony stimulating factor and erythropoietic growth factors should not be administered as prophylaxis for Cycle 1 but may be used in subsequent cycles. The dose of capecitabine must be reduced by two dose levels with the first episode of febrile neutropenia.

6.2.1.2 Dose modification for non-hematologic toxicities

6.2.1.2.1 Dose modification for toxicities common to both sorafenib and capecitabine

Dermatologic toxicities (eg, HFSR, rash), gastrointestinal toxicities (eg, diarrhea), and fatigue are common to both sorafenib and capecitabine. Therefore, because it may not be possible to identify the causative agent should these events present in this trial, a pragmatic approach to dose adjustments has been taken to ensure subject safety.

Hand Foot Skin Reaction

Subjects experiencing HFSR should have their signs and symptoms graded according to the system presented in Table c. Other dermatologic toxicities should be graded according to NCI-CTCAE v4.0.

Subjects with discomfort due to HFSR should be treated with topical emollients, low-potency topical steroids, or urea-containing creams (see Section 6.2.1.2.2).

Table c: Grading for Hand-Foot Skin Reaction

	**Grade 1**	**Grade 2**	**Grade 3**
**NCI-CTCAE v4.0 Palmar-plantar erythrodysesthesia syndrome**[a]	Minimal skin changes or dermatitis (eg, erythema, edema, or hyperkeratosis) without pain	Skin changes (eg, peeling, blisters bleeding, edema, or hyperkeratosis) with pain	Severe skin changes (eg, peeling, blisters, bleeding, edema, or hyperkeratosis) with pain
**Further description / examples of skin changes**	Numbness, dysesthesia/paresthesia, tingling, painless swelling, or erythema of the hands and/or feet	Painful erythema and swelling of the hands and/or feet	Moist desquamation, ulceration, blistering, or severe pain of the hands and/or feet
**Effect on activities**	Does not disrupt normal activities	Limiting instrumental activities of daily life (eg, preparing meals, shopping for groceries or clothes, using the telephone, managing money)	Limiting self-care activities of daily life (eg, bathing, dressing and undressing, feeding self, using the toilet, taking medications) and not bedridden

a: Palmer-planter erythrodysesthesia syndrome is a disorder characterized by redness, marked discomfort, swelling, and tingling in the palms of hands or the soles of the feet.

The dose-modification schedule outlined below (table d) and in Table should be followed as appropriate based on (i) the grade of the toxicity(ies), (ii) the incidences of skin toxicity (including rash and HFSR), gastrointestinal toxicity, and fatigue, and (iii) the cycle of treatment.

All dose modifications will follow the predefined dose levels presented in table a.

Once a dose-reduction modification has been made, NO dose re-escalation will be allowed.

CONFIDENTIAL

This document is confidential. Do not disclose or use except as authorized.

Table d : General guidelines for dose modification for toxicities common to both sorafenib and capecitabine

Grade 1:	If any dermatologic toxicities, gastrointestinal toxicities or fatigue occur at grade 1, maintain doses of capecitabine and sorafenib/placebo. No dose modification is required for any occurrence of Grade-1 fatigue, dermatologic toxicity or gastrointestinal toxicity. The investigator should use symptomatic treatment to alleviate the toxicity (see table e.)
Grade 2 or Grade 3:	If dermatologic toxicities, gastrointestinal toxicities, or fatigue occur at Grade 2 or Grade 3, both agents (ie, capecitabine and sorafenib/placebo) should be held until the toxicity resolves to Grade 1 or less.<br><br>The algorithm in Tabled. should be followed for dose modifications when re-starting study treatment. This algorithm should also be used for each recurrence of these toxicities to determine which drug (ie, capecitabine or sorafenib/placebo) should be reduced. Actions to be taken at each occurrence are outlined below.<br><br>At the first occurrence of Grade 2 or Grade 3 fatigue, dermatologic toxicity, and/or gastrointestinal toxicity, the dose of capecitabine should be reduced by one dose level when restarting study treatment (see table a). Sorafenib/placebo should be restarted at the same dose as prior to the onset of the event(s).<br><br>At the second occurrence of Grade 2 or Grade 3 fatigue, dermatologic toxicity, and/or gastrointestinal toxicity, the dose of sorafenib/placebo should be reduced by one dose level when restarting study treatment (see table a.). Capecitabine should be restarted at the same dose as prior to the onset of the event(s).<br><br>As a general rule, at any subsequent occurrence of Grade 2 or Grade 3 fatigue, dermatologic toxicity, and/or gastrointestinal toxicity, if the last dose modification was made for sorafenib/placebo, sorafenib/placebo should be restarted at the same dose as prior to the onset of the event(s), and the dose of capecitabine should be reduced by one dose level. If, on the other hand, the last dose modification was made for capecitabine, capecitabine should be restarted at the same dose as prior to the onset of the event(s), and the dose sorafenib/placebo should be reduce by one dose level.<br><br>Subjects who continue to experience toxicity and require a dose reduction below the lowest dose level of capecitabine (500 mg/m^2) or sorafenib/ placebo (200 mg/1 placebo tablet twice daily every other day) must discontinue study drug.
Grade 4 (GI toxicities):	Grade 4 toxicities require both study drugs to be discontinued permanently.

Table e: Recommended Dose Modifications for Fatigue, Dermatologic Toxicities and/or GI Toxicities

Grade 1
**No interruption of study drugs or dose reductions of study drugs are required for Grade-1 adverse events.**

Grade 2 or Grade 3 - FIRST occurrence				
If dose at onset of event is:		**Then:**	When event resolves, resume treatment at:	
Sorafenib/placebo	200 mg/1 tablet in the am and 400 mg/ 2 tablets in the pm<br>**OR**[a]<br>400 mg/ 2 tablets twice daily	Hold both sorafenib/placebo and capecitabine until the AE has resolved to Grade 1 or less then resume treatment as directed.[b]	**Sorafenib/placebo**<br>Same dose as prior to event	**200 mg/1 tablet in the am and 400 mg/ 2 tablets in the pm**<br>OR[a]<br>**400 mg/2 tablets twice daily**
Capecitabine	1,000 mg/m^2 twice daily<br>**OR**[a]<br>1,250 mg/m^2 twice daily		**Capecitabine**<br>Reduce dose	**750 mg/m^2 twice daily**<br>OR[a]<br>**1,000 mg/m^2 twice daily**

Grade 2 or Grade 3 - SECOND occurrence				
If dose at onset of event is:		**Then:**	When event resolves, resume treatment at:	
Sorafenib/ placebo	200 mg/1 tablet in the am and 400 mg/ 2 tablets in the pm, daily<br>**OR**[a]<br>400 mg/ 2 tablets twice daily	Hold both sorafenib/placebo and capecitabine until the AE has resolved to Grade 1 or less then resume treatment as directed.[b]	**Sorafenib/ placebo**<br>Reduce dose	**200 mg/1 tablet twice daily**<br>OR[a]<br>**200 mg/1 tablet in the am and 400 mg/ 2 tablets in the pm, daily**
Capecitabine	750 mg/m^2 twice daily<br>**OR**[a]<br>1,000 mg/m^2 twice daily		**Capecitabine**<br>Same dose as prior to event	**750 mg/m^2 twice daily**<br>OR[a]<br>**1,000 mg/m^2 twice daily**

a: The dose at which study drug is resumed is based on the dose being administered at the onset of the AE and must follow the predefined dose levels as specified in table Error! Reference source not found.a. (sorafenib/capecitabine).

b: Interruption in administration of either study drug for more than 21 days requires permanent discontinuation of that study drug.

Table e: Recommended Dose Modifications for Fatigue, Dermatologic Toxicities and/or GI Toxicities

Grade 2 or Grade 3 – THIRD and all SUBSEQUENT occurrences					
If dose at onset of event is:			**Then:**	When event resolves, resume treatment at:	
A	**Sorafenib/placebo**	200 mg/ 1 tablet twice daily<br>**OR**[a]<br>200 mg/ 1 tablet in the am and 400 mg/ 2 tablets in the pm, daily	Hold both sorafenib/placebo and capecitabine until the AE has resolved to Grade 1 or less then resume treatment as directed.[b]	**Sorafenib/placebo**<br>Same dose as prior to event	**200 mg/ 1 tablet twice daily**<br>OR[a]<br>**200 mg/ 1 tablet in the am and 400 mg/ 2 tablets in the pm, daily**
	**Capecitabine**	750 mg/m^2 twice daily<br>**OR**[a]<br>1,000 mg/m^2 twice daily		**Capecitabine**<br>Reduce dose	**500 mg/m^2 twice daily**<br>OR[a]<br>**750 mg/m^2 twice daily**
B	**Sorafenib/placebo**	200 mg/ 1 tablet twice daily<br>**OR**[a]<br>200 mg/ 1 tablet in the am and 400 mg/ 2 tablets in the pm, daily	Hold both sorafenib/placebo and capecitabine until the AE has resolved to Grade 1 or less then resume treatment as directed.[b]	**Sorafenib/placebo**<br>Reduce dose	**200 mg/ 1 tablet twice daily, every other day**<br>OR[a]<br>**200 mg/ 1 tablet twice daily**
	**Capecitabine**	500 mg/m^2 twice daily<br>**OR**[a]<br>750 mg/m^2 twice daily		**Capecitabine**<br>Same dose as prior to event	**500 mg/m^2 twice daily**<br>OR[a]<br>**750 mg/m^2 twice daily**
C	**Sorafenib/placebo**	200 mg/ 1 tablet twice daily	Hold both sorafenib/placebo and capecitabine until the AE has resolved to Grade 1 or less then resume treatment as directed.[b]	**Sorafenib/placebo**<br>Same dose as prior to event	**200 mg/ 1 tablet twice daily**
	**Capecitabine**	750 mg/m^2 twice daily		**Capecitabine**<br>Reduce dose	**500 mg/m^2 twice daily**
D	**Sorafenib/placebo**	200 mg/ 1 tablet twice daily, every other day	Hold both sorafenib/placebo and capecitabine until the AE has resolved to Grade 1 or less then resume treatment as directed.[b]	**Sorafenib/placebo**<br>Same dose as prior to event	**200 mg/ 1 tablet twice daily, every other day**
	**Capecitabine**	750 mg/m^2 twice daily		**Capecitabine**<br>Reduce dose	**500 mg/m^2 twice daily**
E	**Sorafenib/placebo**	200 mg/ 1 tablet twice daily, every other day	Discontinue both drugs permanently	—	
	**Capecitabine**	500 mg/m^2 twice daily			

Grade 4 (GI toxicities)
**Grade 4 toxicities require both study drugs to be discontinued permanently.**

a: **The dose at which study drug is resumed is based on the dose being administered at the onset of the AE and must follow the predefined dose levels as specified in** table **a.(sorafenib/capecitabine).**

b: **Interruption in administration of either study drug for more than 21 days requires permanent discontinuation of that study drug.**

CONFIDENTIAL

This document is confidential. Do not disclose or use except as authorized.

6.2.1.2.2   Prevention/management strategies for hand-foot-skin reaction

Recommended prevention/management strategies for skin toxicities consistent with HFSR are summarized in Table f.

Table f. Recommended Prevention/Management Strategies for Skin Toxicities Consistent with Hand-Foot-Skin-Reaction

Toxicity Grade	Practical Prevention / Management Strategies for HFSR
**Grade 0 (Preventive strategies)**	• Maintain frequent contact with trial physician to ensure early diagnosis of HFSR.<br>• Practical prevention strategies<br>o Pedicure[a] for subjects with pre-existing hyperkeratosis.<br>o Subjects should avoid hot water, and clothing or activities that can cause friction on the skin.<br>o Moisturizing cream should be applied sparingly.<br>• Padded gloves and open shoes with padded soles should be worn to relieve pressure points.
**Grade 1 Any occurrence**	• Continue preventive strategies and in addition:<br>o Soak hands in cool water.<br>o Apply petroleum jelly to moist skin.<br>• In the case of hyperkeratotic lesions, exfoliate the hands or feet and apply moisturizing cream immediately afterwards.
**Grade 2 Any occurrence or Grade 3 Any occurrence**	• Continue supportive/management measures and add analgesic(s) for pain.

a: Pedicure should be done by a podiatrist.

6.2.1.2.3   Prevention/management strategies for diarrhea and fatigue

Diarrhea and fatigue are common side effects of both sorafenib and capecitabine. The same dose-modification algorithm used for skin toxicities (table f) can be used to address these toxicities. However, the preventive/management strategies for diarrhea and fatigue should be consistent with local standards (eg, anti-diarrheals and optimized hydration status for diarrhea).

6.2.1.3   Dose modification and management of sorafenib-specific toxicities

Sorafenib/placebo dose modifications or delays will not impact capecitabine therapy. Subjects who require a delay or dose modification of sorafenib/placebo should continue to receive capecitabine as scheduled.

CONFIDENTIAL
This document is confidential. Do not disclose or use except as authorized.

Treatment-emergent hypertension

Hypertension is a known and potentially serious AE associated with sorafenib treatment. Subjects will undergo brief physical examinations, including blood pressure monitoring, on a weekly basis through the first 6 weeks of therapy. Thereafter, blood pressure will be monitored on Day 1 of each cycle.

Blood pressure measurements that are out of the normal range must be reported by the treating physician to the regional medical monitor/sponsor. Blood pressure measurements considered out of the normal range are diastolic pressure > 90 mm Hg and/or systolic pressure > 140 mm Hg, or a ≥ 20 mm Hg increase in diastolic pressure if the previous measurement was within normal limits.

The dose-modification schedule to be followed in the event of treatment-emergent hypertension is outlined in Tableg. The choice of anti-hypertensive medication to be used in cases of treatment-emergent hypertension will be at the investigator's discretion and based on site-specific treatment guidelines as applicable. All anti-hypertensive medications used for the management of treatment-emergent hypertension should be recorded in the subject’s eCRF.

Once a dose-reduction modification has been made for treatment-emergent hypertension, NO dose re-escalation will be allowed.

CONFIDENTIAL

This document is confidential. Do not disclose or use except as authorized.

Table g.: Management of Treatment-Emergent Hypertension

Grade of Event (NCI-CTCAE v4.0)	Management/ Next Dose
**Grade 1**	Consider increasing blood pressure monitoring. Continue sorafenib dosing as scheduled.
**Grade 2** asymptomatic and diastolic pressure 90-99 mm Hg	Begin anti-hypertensive therapy. Continue sorafenib/placebo dosing as scheduled.
**Grade 2** (symptomatic/persistent)<br>**OR**<br>**Grade 2** symptomatic increase by > 20 mm Hg (diastolic) or to > 140/90 mm Hg if previously within normal limits<br>**OR Grade 3**	Sorafenib/placebo should be held[a] until symptoms resolve <u>and</u> diastolic blood pressure < 90 mm Hg; also treat subject with anti-hypertensives and when sorafenib/placebo is restarted, reduce by 1 dose level.[b]<br>If diastolic blood pressure is not controlled (< 90 mm Hg) on anti-hypertensive therapy, reduce another dose level.[b]
**Grade 4**	Discontinue study drugs

a: Subjects requiring a delay of > 21 days should discontinue sorafenib/placebo unless, in the opinion of the treating physician, the subject may benefit from continued treatment.

b: Subjects requiring dose reductions beyond 200 mg (1 placebo tablet) twice daily, every other day, should discontinue sorafenib/placebo.

6.2.1.4 Dose modification and management of capecitabine-specific toxicities

If the following toxicities occur, they will be deemed to be primarily related to capecitabine. Such toxicities, therefore, warrant specific dose modifications for capecitabine only as described here and in Tableh.

Once the capecitabine dose has been reduced, it **may not** be re-escalated. Doses of capecitabine omitted for toxicity should not be replaced or restored and the subject should resume the planned treatment cycle.

Subjects who require a delay or dose modification of capecitabine should continue to receive sorafenib/placebo as scheduled.

1) Stomatitis (Grade 2 or higher)

If Grade 2 or 3 stomatitis occurs, administration of capecitabine should be immediately interrupted until the event resolves to Grade 1 or less. The subject should be treated symptomatically. Subsequent doses of capecitabine should be administered in accordance with the algorithm in Tableh.

2) Cardiac toxicity

Subjects with cardiac toxicity greater than Grade 2, which is attributable to capecitabine, will be permanently discontinued from capecitabine therapy and withdrawn from study treatment .

3) Dihydropyrimidine dehydrogenase deficiency

If a subject develops clinical manifestations consistent with suspected DHPD deficiency, including Grade 3 or 4 neutropenia, mucositis, diarrhea, and/or encephalopathy, within the first or second cycle of study treatment, the subject should be tested for DHPD levels or genetic polymorphisms if such testing is locally available. If DHPD deficiency is confirmed, the subject should be permanently discontinued from capecitabine and withdrawn from the trial. If such testing is not available and DHPD deficiency is suspected, the subject should be permanently discontinued from capecitabine and withdrawn from study treatment (see Section **Error! Reference source not found.**).

Table h: Dose Modification for Stomatitis, Cardiac Toxicity and DHPD Deficiency Associated With Capecitabine

	Grade 2	Grade 3	Grade 4
**First occurrence**	Interrupt treatment until resolved to Grade 0-1, then continue at same dose with prophylaxis where possible.	Interrupt treatment until re-solved to Grade 0-1, then continue at 75% of the origi-nal dose.	Discontinue permanently.
**Second occur-rence**	Interrupt treatment until resolved to Grade 0-1, then continue at 75% of the original dose.	Interrupt treatment until re-solved to Grade 0-1, then continue at 50% of the origi-nal dose.	Discontinue permanently.
**Third occurrence**	Interrupt treatment until resolved to Grade 0-1, then continue at 50% of the original dose.	Discontinue permanently.	—
**Fourth occurrence**	Discontinue permanently.	—	—

Note: For Grade-1 toxicity, maintain current dose of capecitabine. No dose interruption or modification is required.

As published in the Xeloda US package insert Feb 2010; Appendix **Error! Reference source not found.**).

6.2.1.5 Dose modification for other non-hematologic toxicities (excluding fatigue, der-matologic toxicities, gastrointestinal toxicities, hypertension and toxicities at-tributable to capecitabine)

For other non-hematologic toxicities (excluding fatigue, dermatologic toxicities, gastrointestinal toxicities, hypertension and toxicities attributable to capecitabine), dose modifications are to be handled as outlined in Tablem.

Once a dose-reduction modification has been made for any study drug (sorafenib/placebo or capecitabine), NO dose re-escalation will be allowed.

Table m: Dose Modification for Other Non-Hematologic Toxicities (Excluding Fatigue, Dermatologic Toxicities, Gastrointestinal Toxicities, Hypertension and Toxicities Attributable to Capecitabine)

Toxicity Grade	Occurrence	Sorafenib/Placebo		Capecitabine	
		**Dose Interruption**	**Dose Reduction**	**Dose Interruption**	**Dose Reduction**
**Grade 1**	Any	None	None	None	None
**Grade 2**	First	None	None	Delay until resolved to Grade 1 or less	Resume at full dose
	Subsequent	None	None	Delay until resolved to Grade 1 or less	Reduce dose by one dose level[a,b]
**Grade 3**	Any	Delay until resolved to Grade 1 or less	Reduce dose by one dose level[a,b]	Delay until resolved to Grade 1 or less	Reduce dose by one dose level[a,b]
**Grade 4**	Any	Discontinue study drug	—	Discontinue study drug	—

a: If recovery is not achieved after 21 days of interruption, study drug should be discontinued.

b: Subjects who continue to experience toxicity and require a dose reduction below the lowest dose level of capecitabine (500 mg/m^2) or sorafenib/placebo (200 mg/1 placebo tablet twice daily, every other day) must discontinue study drug.

7. Correlative/special studies

7.1 Early FDG-PET/CT

In a randomized trial, patients with mCRC received irinotecan-based combination chemotherapy. FDG-PET was carried out before treatment and after two cycles in 51 patients at two centers. Changes in tumor FDG uptake were compared with radiological response after four and eight cycles. There was a strong correlation between metabolic response (changes in SUV) and objective response ($r = 0.57$, $p = 0.00001$), with a sensitivity of 77% and a specificity of 76%. However, there was no significant correlation between metabolic response and time to progression ($p = 0.5$) or overall survival ($p = 0.1$). (Byström 2009).

Serial FDG-PET/CT at D14 of neoadjuvant chemotherapy has already been used to prospectively define the therapeutic strategy in adenocarcinoma of the esophagogastric junction, with major histopathologic remissions being observed exclusively in metabolic responders (Lordick 2007).

In a prospective study, Belgian investigators have been able to show at interim analysis in the first 28 patients that serial FDG-PET/CT is able to identify at D14 patients unlikely to experience a tumor RECIST-based objective response under therapy (Hendlisz 2009).

The hypothesis for this study is that non-response in early FDG-PET/CT will be able to predict worse PFS and OS for the study population under the study regimen.

FDG-PET/CT needs standardization to be comparable across centers. For this study, a central core lab will be responsible to harmonize and homogenize data from different centers.. Standard operating procedures will be used to minimize intra- and inter-patient technical variability.

7.2 Growth modulation index (GMI)

The reader is referred to the "correlative studies background" section for more information about GMI. For this study, the TTP of the patients under study will be compared to the retrospectively collected TTP under their latest prior respective treatment. The TTP2/TTP1 ratio will be calculated and correlated with PFS and OS. The hypothesis is that GMI ratios over 1.33 (or, alternatively, over 1.0) will be able to predict increased PFS and OS.

8. Study calendar

Baseline evaluations, including FDG-PET/CT are to be conducted within 1 week prior to start of protocol therapy. CT scans must be done no more than 4 weeks prior to the start of therapy. In the event that the participant's condition is deteriorating, laboratory evaluations should be repeated within 48 hours prior to initiation of the next cycle of therapy.

All assessments must be performed prior to administration of any study medication. All study assessments and medications should be administered within 3 days of the protocol-specified date, unless otherwise noted.

	Pre-study	Cycle 1	Mid-cycle 1	Cycle 2	Cycle 3	Cycle 4	Cycle 5	Cycle 6	Post-treatment (every six weeks)
Study agents		x		x	x	x	x	x	
Informed consent	x								
History	x	x	x	x	x	x	x	x	x
Physical exam, vital signs	x	x	x	x	x	x	x	x	x
cardiac US	x				x			x	
Lab tests	x	x		x	x	x	x	x	x
Performance sta-tus	x	x	x	x	x	x	x	x	x
FDG-PET/CT		x		x*					
CT scan	x				x*			x*	x*

*CTScan will be performed just before the mentioned cycle

9. Measurement of effect

Although response is not the primary endpoint of this trial, participants with measurable disease will be assessed by RECIST criteria version 1.1 (Eisenhauer 2009). For the purposes of this study, participants should be reevaluated every 6 weeks.

9.1 Definitions

Evaluable for toxicity. All participants who receive at least one dose of study treatment will be evaluable for toxicity from the time of their first treatment.

Evaluable for objective response. Only those participants who have measurable disease present at baseline, have received at least one cycle of therapy, and have had their disease re-evaluated will be considered evaluable for response. These participants will have their response classified according to the definitions stated below. (Note: Participants who exhibit objective disease progression or die prior to the end of cycle 1 will also be considered evaluable.)

Overall survival (OS) is defined as the duration of time from registration in the study until death from any cause or end of follow-up (censoring). For the analysis of survival duration according to early PET response status, the time zero for analyzing OS will however be the date of the second PET examination.

Progression-Free Survival (PFS) is defined as the duration of time from the registration in the study until objective disease progression or death.

9.2 Methods of evaluation of measurable disease

All measurements should be taken and recorded in metric notation, using a ruler, calipers, or digital measurement tool. All baseline evaluations should be performed as closely as possible to the beginning of treatment and never more than 4 weeks before the beginning of treatment.

The same method of assessment and the same technique should be used to characterize each identified and reported lesion at baseline and during follow-up. Imaging-based evaluation is preferred to evaluation by clinical examination when both methods have been used to assess the anti-tumor effect of the treatment.

Clinical lesions. Clinical lesions will only be considered measurable when they are superficial (e.g. skin nodules and palpable lymph nodes). For the case of skin lesions, documentation by color photography, including a ruler to estimate the size of the lesion, is recommended.

Computed tomography (CT). CT should be performed with cuts of 10mm or less in slice thickness contiguously. Spiral CT should be performed using a 5mm contiguous reconstruction algorithm.

9.3 ***FDG-PET(CT).***

The practical guidelines for FDG PET/CT imaging (activity injected; acquisition timing; processing; image analysis; PET/CT data form input) are specified in the Standard Procedure Imaging Manual (SPIM) for PET/CT (see attachment) following as close as possible the EANM procedure guidelines for tumour PET imaging: version1.0 (ref Boellaard et al 2010)

9.4 Other Response Parameters

FDG PET(CT) response will be assessed using the EORTC criteria : a lesion showing at least a 15% reduction of the FDG uptake is considered as responding. Patients will be categorized in 5 classes : (I) all baseline lesions show a response; (II) the majority of lesions show a significant metabolic response; (III) the majority of lesions do NOT show a significant metabolic response; (IV) all lesions do not show a significant response; (V) at least one lesion shows

CONFIDENTIAL
This document is confidential. Do not disclose or use except as authorized.

a significant (>15%) increase of the FDG uptake. For the primary analysis, classes I and II will be considered as responding, and classes III, IV, V as non-responding patients.

The response as defined is undetermined in case of an even number of lesions and 50% of them responding and 50% of them non responding.

10. Adverse event reporting requirements

10.1 Definitions

10.1.1 Adverse event (AE)

An adverse event (AE) is any undesirable sign, symptom or medical condition or experience that develops or worsens in severity after starting the first dose of study treatment or any procedure specified in the protocol, even if the event is not considered to be related to the study.

Clinically relevant abnormal results of diagnostic procedures, including abnormal laboratory findings (eg requiring unscheduled diagnostic procedures or treatment measures, or resulting in withdrawal from the study) are considered as adverse events.

10.1.2 Serious adverse event (SAE)

A serious adverse event (SAE) is any adverse event, occurring at any dose and regardless of causality that:

- Results in death
- Is life-threatening. Life-threatening means that the person was at immediate risk of death from the reaction as it occurred, i.e., it does not include a reaction which hypothetically might have caused death had it occurred in a more severe form.
- Requires or prolongs inpatient hospitalization (i.e., the event required at least a 24-hour hospitalization or prolonged a hospitalization beyond the expected length of stay). Hospitalization admissions or surgical operations scheduled to occur during the study period, but planned prior to study entry are not considered SAEs if the illness or disease existed before the person was enrolled in the trial, provided that it did not deteriorate in an unexpected manner during the trial (e.g., surgery performed earlier than planned).
- Results in persistent or significant disability. Disability is defined as a substantial disruption of a person’s ability to conduct normal life functions.
- Is an important medical event when, based upon appropriate medical judgment, it may jeopardize the participant and require medical or surgical intervention to prevent one of the outcomes listed above. Examples of such medical events include allergic bron-

chospasm requiring intensive treatment in an emergency room or at home; blood dyscrasias or convulsions that do not result in inpatient hospitalization, or the development of drug dependency or drug abuse.

- Is a congenital abnormality/birth defect.

Events *not* considered to be serious adverse events are hospitalizations for:

- routine treatment or monitoring of the studied indication, not associated with any deterioration in condition, or for elective procedures
- elective or pre-planned treatment for a pre-existing condition that did not worsen
- emergency outpatient treatment for an event not fulfilling the serious criteria outlined above and not resulting in inpatient admission
- respite care

10.1.3 Expectedness

Adverse events can be 'Expected' or 'Unexpected.'

10.1.3.1 Expected adverse event

Expected adverse events are those that have been previously identified as resulting from administration of the agent. For the purposes of this study, an adverse event is considered expected when it appears in the current adverse event list, the package insert or is included in the informed consent document as a potential risk.

Refer to the appropriate for a listing of expected adverse events associated with the study agents.

10.1.3.2 Unexpected adverse event

For the purposes of this study, an adverse event is considered unexpected when it varies in nature, intensity or frequency from information provided in the current adverse event list, the package insert or when it is not included in the informed consent document as a potential risk.

10.1.4 Association with the use of the study treatment

An adverse event is considered associated with the study treatment use if the attribution is possible, probable or very likely. Attribution will be assigned as follows:

- Very likely – The AE cannot be reasonably explained by an alternative causality.
- Probable – The AE is likely related to the study treatment.
- Possible – The AE may be related to the study treatment.
- Unlikely – The AE is doubtfully related to the study treatment.
- Unrelated – The AE is clearly NOT related to the study treatment.

10.2 Procedures for AE and SAE Recording

Participating investigators will assess the occurrence of AEs and SAEs at all participant evaluations during the study. All AEs and SAEs whether reported by the participant, discovered during questioning, directly observed, or detected by physical examination, laboratory test or other means, will be recorded in the participant's medical record and on the appropriate study-specific case report forms.

The descriptions and grading scales found in the CTEP active version of the NCI Common Terminology Criteria for Adverse Events (CTCAE) will be used for AE reporting. The CTEP Active Version of the CTCAE is identified and located on the CTEP website at: http://evs.nci.nih.gov/ftp1/CTCAE/CTCAE_4.03_2010-06-14_QuickReference_5x7.pdf. All appropriate treatment areas should have access to a copy of the CTEP Active Version of CTCAE.

10.3 Reporting Requirements

The study must be conducted in compliance with national Belgian regulations, European Union regulations, local safety reporting requirements, and reporting requirements of the principal investigator.

Each investigative site will be responsible to report SAEs that occur at that institution to their respective IRB. It is the responsibility of each participating investigator to report serious adverse events to the study sponsor as described below.

It is the responsibility of the principal investigator to report the SAEs to the principal IRB and to Bayer and to report the SUSARs to the CA as described below.

10.4 Reporting to the Study Sponsor

10.4.1 Serious Adverse Event Reporting

All serious adverse events that occur after the initial dose of study treatment, during treatment, or within 30 days of the last dose of treatment must be reported to the Principal Investigator on the SAE form.

Participating investigators must report each serious adverse event to the Principal Investigator within 24 hours of learning of the occurrence. In the event that the participating investigator does not become aware of the serious adverse event immediately (e.g., participant sought treatment elsewhere), the participating investigator is to report the event within 24 hours after learning of it and document the time of his first awareness of the adverse event. Report serious adverse events by telephone, email or fax to:

Dr Alain Hendlisz

Tel.: +32 2 541 31 96

Email: alain.hendlisz@bordet.be ; anne.denis@bordet.be

Fax: +32 2 538 18 11

Within the following 24–48h, the participating investigator must provide follow-up information on the serious adverse event using a specific form to be sent to the principal investigator. Follow-up information should describe whether the event has resolved or continues, if and how the event was treated, and whether the participant will continue or discontinue study participation.

10.4.2 Non-Serious Adverse Event Reporting

Non-serious adverse events will be reported to the Data Center on the toxicity Case Report Forms.

10.5 Reporting to the Institutional Review Board (IRB)

Investigative sites should report serious adverse events to their respective IRB according to the local IRB's policies and procedures in reporting adverse events. A copy of the submitted institutional SAE form should be forwarded to:

Dr Alain Hendlisz

Tel.: +32 2 541 31 96

Email: alain.hendlisz@bordet.be ; anne.denis@bordet.be

Fax: +32 2 538 18 11

10.6 Reporting to the principal Institutional Review Board (IRB)

The Principal Investigator is responsible to report all serious adverse events and the Annual Safety Report (received from Bayer) to the principal IRB.

10.7 Reporting to competent authorities (CA)

The Principal Investigator will submit the Suspected Unexpected Serious Adverse Reactions (SUSARs) and the Annual Safety Report (received from Bayer) to the CA.

10.8 Reporting to Bayer

The Principal Investigator is responsible to report all serious adverse events within 24 hours to Bayer.

BAYER

Fax:02/720.74.33
E-mail: drugsafety.belux@bayer.com

10.9 Monitoring of Adverse Events and Period of Observation

All adverse events, both serious and non-serious, and deaths that are encountered from initiation of study intervention, throughout the study, and within 30 days of the last study intervention should be followed to their resolution, or until the participating investigator assesses them as stable, or the participating investigator determines the event to be irreversible, or the participant is lost to follow-up. The presence and resolution of AEs and SAEs (with dates) should be documented on the appropriate case report form and recorded in the participant's medical record to facilitate source data verification.

For some SAEs, the study sponsor or designee may follow-up by telephone, fax, and/or monitoring visit to obtain additional case details deemed necessary to appropriately evaluate the SAE report (e.g., hospital discharge summary, consultant report, or autopsy report).

Participants should be instructed to report any serious post-study event(s) that might reasonably be related to participation in this study. Participating investigators should notify the Principal Investigator and their respective IRB of any unanticipated death or adverse event occurring after a participant has discontinued or terminated study participation that may reasonably be related to the study.

11. Data and safety monitoring

11.1 Data reporting

The schedule for completion and submission of case report forms to the Data Center is as follows:

Schedule of completion and submission of case report forms

Form	Submission timeline
Eligibility checklist	Complete prior to registration with the Data Center
On study form	Within 14 days of registration
Baseline assessment form	Within 14 days of registration
Treatment form	Within 10 days of the last day of the cycle
Adverse event report form	Within 10 days of the last day of the cycle
Response assessment form	Within 10 days of the completion of the cycle required for response evaluation
Off treatment/off study form	Within 14 days of completing treatment or being taken off study for any reason
Follow up form	Within 14 days of the protocol-defined follow-up visit or call

The Data Center is responsible for compiling data for all participants and for providing the data to the Principal Investigator for review.

11.2 Monitoring

Involvement in this study as a participating investigator implies acceptance of potential audits or inspections, including source data verification, by representatives designated by the Principal Investigator. The purpose of these audits or inspections is to examine study-related activities and documents to determine whether these activities were conducted and data were recorded, analyzed, and accurately reported in accordance with the protocol, institutional policy, Good Clinical Practice (GCP), and any applicable regulatory requirements.

All data will be monitored for timeliness of submission, completeness, and adherence to protocol requirements. Monitoring will begin at the time of participant registration and will continue during protocol performance and completion.

11.3 Annual Safety Reporting

Once a year, a global safety report will be issued and transmitted to the competent authorities by the Principal Investigator. A copy of this report will be also transmitted to the Central Ethical Committee.

12. Regulatory considerations

12.1 Protocol Review and Amendments

This protocol, the proposed informed consent and all forms of participant information related to the study (e.g., advertisements used to recruit participants) and any other necessary documents must be submitted, reviewed and approved by a properly constituted IRB (ethics committee) governing each study location.

Any changes made to the protocol must be submitted as amendments and must be approved by the IRB prior to implementation. Any changes in study conduct must be reported to the IRB. The Principal Investigator will disseminate protocol amendment information to all participating investigators.

All decisions of the IRB concerning the conduct of the study must be made in writing.

12.2 Informed Consent

All participants must be provided a consent form describing this study and providing sufficient information for participants to make an informed decision about their participation in this study. The formal consent of a participant, using the IRB approved consent form, must be obtained before the participant is involved in any study-related procedure. The consent form must be signed and dated by the participant or the participant's legally authorized representative, and by the person obtaining the consent. The participant must be given a copy of the signed and dated consent document. The original signed copy of the consent document must be retained in the medical record or research file.

12.3 Ethics and Good Clinical Practice (GCP)

This study is to be conducted according to the following considerations, which represent good and sound research practice:

- The Declaration of Helsinki
  (www.wma.net/en/30publications/10policies/b3/index.html)

- European Union laws and regulations

- Belgian laws
- Local research policies and procedures

It is understood that deviations from the protocol should be avoided, except when necessary to eliminate an immediate hazard to a research participant. In such case, the deviation must be reported to the IRB according to the local reporting policy.

12.4 Study Documentation

The investigator must prepare and maintain adequate and accurate case histories designed to record all observations and other data pertinent to the study for each research participant. This information enables the study to be fully documented and the study data to be subsequently verified.

Original source documents supporting entries in the case report forms include but are not limited to hospital records, clinical charts, laboratory and pharmacy records, recorded data from automated instruments, microfiches, photographic negatives, microfilm or magnetic media, and x-rays.

12.5 Records Retention

All study-related documents must be retained for the maximum period required by applicable European or national regulations and guidelines or institutional policies.

13. Statistical considerations

Sample size has been estimated in order to be able to test the null hypothesis that the overall survival rate at 6 months is less than 30%. This hypothesis will be tested using a binomial distribution. The study should be able to reject the null hypothesis, using a 1-sided test with $\alpha = 0.025$, with a power of 90% in case of a true overall survival $\geq$ 50% (rate at 6 months). The sample size required is 66 eligible patients (to be followed for 6 months minimum). Analysis will be done on all registered patients using an ITT approach on all eligible patients.

A co-primary endpoint is to compare the overall survival of patients assessed as early PET responders and of patients assessed as early PET non responders (the clinicians will remain blinded for PET response assessment). For this primary analysis, patients who will undergo the second PET assessment will be eligible and time zero for measuring survival will be the date of this second PET examination. It is anticipated that 95% of the patients will be eligible for the analysis with a 50% expected rate of early PET non-responders (result obtained from an unpublished study conducted at Jules-Bordet Institute). With 66 patients registered, we anticipate then that 63 patients will be available for the co-primary endpoint. With 63 patients and our assumption that the HR for the comparison between the survival distributions will be around 0.385 (based on the previously mentioned unpublished study), we will need using a

two-sided logrank test at the 2.5% level (2.5% chosen because of the existence of 2 co-primary endpoints), 54 events (power of 90%). With 63 patients and a follow-up after accrual of 1 year, we should reach this number of 54 events. However, to account for another possible 5% drop-out (patient's refusal for undergoing the second PET examination for instance), sample size should be increased to 70 eligible patients.

Secondary endpoints are to estimate progression-free survival and objective response rate, and to describe the adverse reactions associated with the study regimen in the study population. Also, to determine the correlation of early metabolic response, as assessed by FDG-PET/CT immediately before the first and the second cycles of treatment with the study regimen, with overall survival, progression-free survival, and response rate, and to determine the correlation of *growth modulation index* (GMI), defined as the time to progression under the study regimen over the time to progression under the latest regimen administered to the patient, with overall survival and progression-free survival.

The study is designed as a single-arm phase II study, with all patients accrued in one stage. No early stopping rules will be used.

13.1 Sample Size/Accrual Rate

The expected accrual is 40 patients/year. The duration of follow-up after completion of accrual will be one year. Accrual duration is then expected to be 21 months. Study data should be mature 32 months after accrual start.

13.2 Stratification Factors

Patients will be stratified according to KRAS mutation status. Descriptive analysis will be done for patients with BRAF mutations.

13.3 Analysis of Secondary Endpoints

For most of the other endpoints, no comparative analysis will be carried out. Estimates of theoretical parameters will be provided together with 95% confidence intervals. The rate of patients surviving at 1 year, expecting a 6 months median survival time in the overall population (60 patients), could be estimated with a 95% confidence interval of length around 24%.

13.4 Reporting and Exclusions

13.4.1 *Evaluation of toxicity*. All participants will be evaluable for toxicity from the time of their first treatment.

13.4.2 *Evaluation of response*. All participants included in the study must be assessed for response to treatment, even if there are major protocol treatment deviations or if they are ineligible. Each participant should be assigned one of

the following categories: 1) complete response, 2) partial response, 3) stable disease, 4) progressive disease, 5) early death from malignant disease, 6) early death from toxicity, 7) early death because of other cause, or 9) unknown (not assessable, insufficient data).

14. Investigator authorization procedure

Investigators will be authorized to register a patient in this trial only once they have returned the following documents to Institut Jules Bordet:

- The updated, signed, and dated curriculum vitae of the investigators.

- A commitment statement / study acknowledgment form, stating that the investigator will fully comply with the protocol. This must include an estimate of yearly accrual and a statement on any conflict of interest that may arise due to trial participation.
  A copy of the favourable opinion of the local ethics committee mentioning the documents that were reviewed (including version numbers and dates for all documents). A list of all members of the ethics committee is also required.

- The signature log-list of the staff members with a sample of each authorized signature and the indication of the level of delegations. In case patients receive treatment at a satellite institution, i.e. outside the authorized institution, details on the satellite institution, including the CV of the local investigator, normal lab ranges and the approval of an ethics committee will have to be transmitted to Institut Jules Bordet.

- All applicable legal and regulatory requirements must be fulfilled.

- Patient inclusion from non-authorized centres will not be accepted.

15. Forms and procedures for collecting data

15.1 Case report forms and schedule for completion

Data will be reported on the forms specifically designed for this study. All participants should send forms directly to Institut Jules Bordet. All forms must be dated and signed by the responsible investigator or an authorized staff member.

15.2 Data flow

The case report forms (CRF) must be completed, dated and signed by the investigator or an authorized staff member as soon as the requested information is available (timelines will be speci-

fied on the different forms). Before the start of the study, the list of staff members authorized to sign case report forms (with a sample of their signature) must be sent to the coordinating centre by the responsible investigator. In all cases, it remains the responsibility of the investigator to check that all original case report forms are sent to the coordinating centre and are filled completely and correctly. The investigator must keep copies of all case report forms. The original forms must be immediately returned to the coordinating centre. When satellite institutions are involved, all contacts are done exclusively with the primary institution, for purposes of data collection and all other study related issues. If an investigator (or an authorized staff member) needs to modify a CRF after the original form has been returned to the allocated data centre, he/she should notify the coordinating institution by using the Data Correction Form. The original Data Correction Form should be sent to the coordinating institution and a copy should be kept with the other CRF copies. The investigator's CRF copies must not be modified unless modifications are reported on a Query Form or a Data Correction Form.

There will be a separate PET/CT CRF which will be completed by the investigator of the PET/CT centre to where the patient has been sent. The PET/CT CRF will be sent at Institut Bordet not more than one week after the procedure. This specific CRF will be reviewed and validated by the MICoLab.

16. Quality assurance

16.1 Control of data consistency

Computerized and manual consistency checks will be performed on newly entered forms; queries will be issued in case of inconsistencies. Consistent forms will be validated by the data manager and then entered into the master database. Inconsistent forms will be kept pending until resolution of inconsistencies.

16.2 Audits

To ensure quality of data, study integrity and compliance with the protocol and the various applicable regulations and guidelines, site visits may be conducted to participating institutions. Quality assurance visits by a coordinating institution physicist are planned. The investigator, by accepting to participate in this protocol, agrees to co-operate fully with any quality assurance visit undertaken by third parties, including representatives from the coordinating institution or national regulatory authorities. The investigator will also grant direct access to documentation pertaining to the clinical trial (including CRFs, source documents, hospital patient charts and other study files) to authorized individuals.

17. Ethical considerations

17.1 Patient protection

The principal investigator will ensure that this study conforms to the Declaration of Helsinki (available at http://www.wma.net/e/policy/pdf/17c.pdf) or the laws and regulations of the country, whichever provides the greatest protection of the patient.

The study follows the International Conference on Harmonization E 6 (R1) Guideline for Good Clinical Practice, reference number CPMP/ICH/135/95 (available at http://www.emea.europa.eu/pdfs/human/ich/013595en.pdf).

The competent ethics committees must approve this protocol, as required by the applicable national legislation.

17.2 Subject identification

The name of the patient will neither be asked for nor recorded at the Data Centre. A sequential identification number will be automatically allocated to each patient registered in the trial. This number will identify the patient and must be included on all case report forms. In order to avoid identification errors, the patient's code (maximum of 4 digits), and date of birth will also be reported on the case report forms.

17.3 Informed consent

All patients will be informed about

- the aims of the study
- the possible adverse events
- the procedures and possible hazards to which the patient will be exposed
- strict confidentiality of any patient data
- medical records possibly being reviewed for trial purposes by authorized individuals other than their treating physician

The template of the patient's informed consent statement is given as an appendix to this protocol. The informed consent documents are to be submitted to ethics committees for approval. The competent ethics committee for each institution must approve the informed consent documents before the centre can join the study. It is the responsibility of the competent ethics committee to ensure that the informed documents comply with The International Conference on Harmonisation of Technical Requirements for Registration of Pharmaceuticals for Human Use Good Clinical practice (ICH-GCP) guidelines and all applicable national legislation.

It is emphasized in the patient information sheet that participation is voluntary and that the patient is free to refuse further participation in the protocol whenever he/she wants to. This will not have any impact on the patient's subsequent care. Documented informed consent must be obtained for all patients included in the study before they are registered at the Data Centre. The written informed consent form must be signed and personally dated by the patient or by the patient's legally acceptable representative.

All of the above must be in accordance with the applicable national legislation and local regulatory requirements.

18. Administrative responsibilities

18.1 The Study Coordinator

The Study Coordinator is responsible for

- writing the protocol
- reviewing all case report forms and documenting his/her review on evaluation forms
- discussing the contents of the reports with the Data Manager and the Statistician
- publishing the study results
- answering all clinical questions concerning eligibility, treatment and evaluation of the patients

18.2 The Joint Study Management Team

The Joint Study Management Team, chaired by the Study Coordinator, is responsible for the daily conduct of the Study and is constituted of key supportive collaborators from the Institut Jules Bordet appointed by the Chairperson.

19. Trial sponsorship and financing

A grant from Bayer Belgium Inc to the Institut Jules Bordet provides funding for the study. The Sponsor is Institut Jules Bordet – Centre des Tumeurs de l'ULB, rue Héger-Bordet, 1, 1000 Brussels, represented by Dr. D. de Valeriola (Medical Director Institut Jules Bordet); Mr. P. Goblet (Managing Director Centres des Tumeurs de l'ULB) and Dr. A. Hendlisz (Head of Gastroenterology Unit).

20. Trial insurance

The Sponsor has taken out a liability insurance policy to cover its liability as required by applicable law and especially in accordance with the Belgian Law relating to experiments in

humans dated May 7, 2004, its subsequent amendments and Royal Decree of execution. Upon request, the Sponsor will provide the Investigator with a certificate of insurance. The insurance of the Sponsor does not relieve the Institution and the Investigator of any obligation to maintain their liability insurance policy.

Clinical trial insurance is only valid in centres authorized from the coordinating institution. Clinical trial insurance only covers patients treated at satellite institutions if these satellite institutions are properly reported to the coordinating centre.

21. Publication plan

21.1 Publication and Presentation rules

21.1.1 General Principles

The Sponsor recognizes the Investigator's right of utilizing data derived from the Study for teaching purposes, communication at congresses and scientific publications.

Nevertheless, in order to ensure the accuracy and scientific value of the information, while preserving the independence and accountability of the Investigator and the confidentiality of the information, only clear, checked and validated data shall be used. To that effect, it is essential that the Investigator and the Sponsor exchange and discuss, prior to any publication or communication, any draft publication or communication made by the Investigator.

Therefore, the Investigator undertakes and shall cause any sub-Investigators, not to make any publication, communication or release pertaining to the results of the Study, without the prior consent of the Sponsor. The Investigator shall send to the Sponsor a copy of the manuscript for review and possible comments at least forty-five (45) calendar days in advance of the date of submission to the journal and at least twenty (20) calendar days in advance for abstracts. The publication shall be delayed until approval of publication is given in writing by the Sponsor, it being understood that the Sponsor cannot refuse its consent without reasonable cause. The Investigator agrees to include the modifications requested by the Sponsor, provided they do not jeopardize the accuracy and/or the scientific value of the publication. In the event of any disagreement in the content of any publication, both the Investigator's and Sponsor's opinion shall be fairly and sufficiently represented in the publication. The absence of answer at the end of the above-mentioned twenty (20) / forty-five (45) days deadline, depending on the fact that it concerns an abstract or not, is automatically equivalent to a negative response of the Sponsor as for the question of the publication of the document.

Shall the Sponsor desire to protect by a property right any Information contained in the publication, it has the right to postpone the publication, for a period not to exceed twelve (12) months.

CONFIDENTIAL
This document is confidential. Do not disclose or use except as authorized.

In multicenter studies, the Investigator agrees not to publish the results of the Study on his/her Site before the results of the multicenter Study are published. If no publication has occurred within twelve (12) months of the database lock, the Investigator shall have the right to publish independently the results of this Study on his/her Site, subject to the review procedure set forth herein. However, in a multicentre study based on the collaboration of many centres, any publication of results must acknowledge all centres.

The Investigator / Institution shall not use the name(s) of the Sponsor and/or of its employees in advertising or promotional material or publication without the prior written consent of the Sponsor. The Sponsor shall not use the name(s) of the Investigator / Institution in advertising or promotional material or publication without having received his/her and/or their prior written consent(s).

The Sponsor has the right at any time to publish the results of the Study.

This restriction of publications and communications shall remain in effect during ten (10) years after the termination of this Study.

The Study Coordinator will write the final publication of the trial results, based on the final analysis. Within six months from the final analysis of the core protocol, the Study Coordinator will submit a draft manuscript to the participating institutions for review and revision. After review by all co-authors, he will submit the manuscript to a scientific journal. Parts of the study may be presented to relevant scientific meetings.

21.1.2 Authorship

The Study Coordinator shall be author of all and any publication and presentation.

Prime authorship position of primary endpoints related publications/presentations shall be given to the Study Coordinator and last authorship position shall be given to Patrick Flamen, MD, PhD.

Prime authorship position of secondary endpoints related publications / presentations shall be given as follows:

- to Patrick Flamen, MD, PhD for functional imaging (PET/CT)-related publications / presentations;

Last authorship position of secondary endpoints-related publications / presentations shall be given to the Study Coordinator.

Other authorship positions shall be given to those who have provided the most scientific leadership (e.g. clinical/translational/bio statistical expertise related to study hypotheses, trial design, protocol writing or medical review) rather than those whose contributions have been more supportive (e.g. study management).

Any publication or presentation shall mention the statistician responsible for the related analysis among the authors.

Other authorship positions shall be given to:

- the highest recruiting centres (in the name of the Investigator);
- individuals involved in the central trial management;
- young team members (e.g. fellows, PhD, Post Graduated) contributing significantly to the trial;
- any other trial partner not listed above.

All manuscripts will include an appropriate acknowledgment section, mentioning all investigators who have contributed to the trial, the staff of the Data Centre involved in the study, as well as the supporting bodies. The number of acknowledgments per participating entity shall depend on the journal's rules and be based on fair and practical considerations.

Authorship for abstracts or presentations will be determined according to the same criteria, although relevant guidelines for the number of authors must be respected.

22. References

(Awada submitted) Awada A, Gil T, Whenham N, Van Hamme J, Mancini I, Besse-Hammer T, Brendel X, Delesen H, Joosten MC, Lathia CD, O'Leary J, Loembé BA, Piccart-Ghebart M, Hendlisz A. Safety and Pharmacokinetics of Sorafenib Combined with Capecitabine in Patients with Advanced Solid Tumors: Results of a Phase I Trial (Submitted for publication).

(Baselga 2009) Baselga J, Segalla JGM, Roché H, del Giglio A, Ciruelos EM, Cabral Filho S, Gomez P, Lluch A, Llombart A, Costa F. SOLTI-0701: A double-blind, randomized phase 2b study evaluating the efficacy and safety of sorafenib (SOR) compared to placebo (PL) when administered in combination with capecitabine (CAP) in patients (pts) with locally advanced (adv) or metastatic (met) breast cancer (BC). ESMO/ECCO 2009: Abs. 3LBA.

(Bonetti 2001) Bonetti A, Zaninelli M, Leone R, Franceschi T, Fraccon AP, Pasini F, Sabbioni R, Cetto GL, Sich D, Brienza S, Howell SB. Use of the ratio of time to progression following first- and second-line therapy to document the activity of the combination of oxaliplatin with 5-fluorouracil in the treatment of colorectal carcinoma. Ann Oncol. 2001;12:187-91.

(Buyse 2000) Buyse M, Thirion P, Carlson RW, Burzykowski T, Molenberghs G, Piedbois P. Relation between tumour response to first-line chemotherapy and survival in advanced colorectal cancer: a meta-analysis. Meta-Analysis Group in Cancer. Lancet. 2000;356:373-8.

(Byström 2009) Byström P, Berglund A, Garske U, Jacobsson H, Sundin A, Nygren P, Frödin JE, Glimelius B. Early prediction of response to first-line chemotherapy by sequential [18F]-2- fluoro-2-deoxy-D-glucose positron emission tomography in patients with advanced colorectal cancer. Ann Oncol. 2009;20(6):1057-61.

(Cao 2009) Cao Y, Zhong W, Sun Y. Improvement of antiangiogenic cancer therapy by understanding the mechanisms of angiogenic factor interplay and drug resistance. Semin Cancer Biol. 2009;19:338-43.

(Cassidy 2007) Cassidy J, Saltz L. Bevacizumab (Bev) in combination with XELOX or FOLFOX4: Updated efficacy results from XELOX-1/ NO16966, a randomized phase III trial in firstline metastatic colorectal cancer. 2007 ASCO annual meeting. Abs 4028, 4030.

(Comella 2002) Comella P, Casaretti R, Crucitta E, De Vita F, Palmeri S, Avallone A, Orditura M, De Lucia L, Del Prete S, Catalano G, Lorusso V, Comella G. Oxaliplatin plus raltitrexed and leucovorin-modulated 5-fluorouracil i.v. bolus: a salvage regimen for colorectal cancer patients. Br J Cancer. 2002;86:1871-5.

(Cunningham 2004) Cunningham D, Humblet Y, Siena S, Khayat D, Bleiberg H, Santoro A, Bets D, Mueser M, Harstrick A, Verslype C, Chau I, Van Cutsem E. Cetuximab monotherapy and cetuximab plus irinotecan in irinotecan-refractory metastatic colorectal cancer. N Engl J Med. 2004 Jul 22;351:337-45.

(Eisenhauer 2009) Eisenhauer EA, Therasse P, Bogaerts J, Schwartz LH, Sargent D, Ford R, Dancey J, Arbuck S, Gwyther S, Mooney M, Rubinstein L, Shankar L, Dodd L, Kaplan R, Lacombe D, Verweij J. New response evaluation criteria in solid tumours: revised RECIST guideline (version 1.1). Eur J Cancer. 2009;45:228-47.

(Falcone 2007) Falcone A, Ricci S, Brunetti I et al. Phase III trial of infusional fluorouracil, leucovorin, oxaliplatin, and irinotecan (FOLFOXIRI) compared with infusional fluorouracil, leucovorin, and irinotecan (FOLFIRI) as first-line treatment for metastatic colorectal cancer: the Gruppo Oncologico Nord Ovest. J Clin Oncol. 2007;25:1670-6.

(Fuchs 2007) Fuchs CS, Marshall J, Mitchell E et al. Randomized, controlled trial of irinotecan plus infusional, bolus, or oral fluoropyrimidines in first-line treatment of metastatic colorectal cancer: results from the BICC-C Study. J Clin Oncol. 2007;25:4779-86.

(Gambhir 2002) Gambhir SS. Molecular imaging of cancer with positron emission tomography. Nat Rev Cancer. 2002;2:683-93.

(Giantonio 2007) Giantonio B, Catalano P, Meropol N et al. Bevacizumab in Combination With Oxaliplatin, Fluorouracil, and Leucovorin (FOLFOX4) for Previously Treated Metastatic Colorectal Cancer: Results From the Eastern Cooperative Oncology Group Study E3200 J Clin Oncol. 2007;25:1539-44.

(Goldberg 2007) Goldberg RM, Rothenberg ML, Van Cutsem E, Benson AB 3rd, Blanke CD, Diasio RB, Grothey A, Lenz HJ, Meropol NJ, Ramanathan RK, Becerra CH, Wickham R, Armstrong D, Viele C. The continuum of care: a paradigm for the management of metastatic colorectal cancer. Oncologist. 2007;12:38-50.

(Grothey 2005) Grothey A, Sargent D. Overall survival of patients with advanced colorectal cancer correlates with availability of fluorouracil, irinotecan, and oxaliplatin regardless of whether doublet or single-agent therapy is used first line. J Clin Oncol. 2005;23:9441-2.

(Grothey 2007) Grothey A, Sugrue M, Hedrick E, et al. Association between exposure to bevacizumab (BV) beyond first progression (BBP) and overall survival (OS) in patients (pts) with metastatic colorectal cancer (mCRC): Results from a large observational study (BRiTE). 2007 ASCO Annual Meeting Abstract No: 4036.

(Hendlisz 2009) Hendlisz A, Emonts P, Covas A, Ameye L, Paesmans M, Castany Prado Maria del Rosario R, Machiels G, Van den Eynde M, Vanderlinden B, Flamen P. Is positron emission tomography (PET) with FDG an early predictor of the RECIST morphological response to chemotherapy in metastatic colorectal cancer patients (mCRC)? J Clin Oncol 27:15s, 2009 (suppl; abstr 2533).

(Hurwitz 2004) Hurwitz H, Fehrenbacher L, Novotny W et al: Bevacizumab plus irinotecan, fluorouracil and leucovorin for metastatic colorectal cancer. N Engl J Med 2004;350:2335-42.

(Johnson 2006) Johnson KR, Ringland C, Stokes BJ, Anthony DM, Freemantle N, Irs A, Hill SR, Ward RL. Response rate or time to progression as predictors of survival in trials of metastatic colorectal cancer or non-small-cell lung cancer: a meta-analysis. Lancet Oncol. 2006;7:741-6.

(Jonker 2007) Jonker DJ, O'Callaghan CJ, Karapetis CS, Zalcberg JR, Tu D, Au HJ, Berry SR, Krahn M, Price T, Simes RJ, Tebbutt NC, van Hazel G, Wierzbicki R, Langer C, Moore MJ. Cetuximab for the treatment of colorectal cancer. N Engl J Med. 2007;357:2040-8.

(Koopman 2007) Koopman M, Antonini NF, Douma J, Wals J, Honkoop AH, Erdkamp FL, de Jong RS, Rodenburg CJ, Vreugdenhil G, Loosveld OJ, van Bochove A, Sinnige HA, Creemers GJ, Tesselaar ME, Slee PH, Werter MJ, Mol L, Dalesio O, Punt CJ. Sequential versus combination chemotherapy with capecitabine, irinotecan, and oxaliplatin in advanced colorectal cancer (CAIRO): a phase III randomised controlled trial. Lancet. 2007;370:135-42.

(Lordick 2007) Lordick F, Ott K, Krause BJ, Weber WA, Becker K, Stein HJ, Lorenzen S, Schuster T, Wieder H, Herrmann K, Bredenkamp R, Höfler H, Fink U, Peschel C, Schwaiger M, Siewert JR. PET to assess early metabolic response and to guide treatment of adenocarcinoma of the oesophagogastric junction: the MUNICON phase II trial. Lancet Oncol. 2007;8:797-805.

(Louvet 2001) Louvet C, de Gramont A, Tournigand C. Correlation between progression free survival and response rate in patients with metastatic colorectal carcinoma. Cancer. 2001;91(11):2033-8.

(Maindrault-Goebel 2004) Maindrault-Goebel F, Tournigand C, André T, Carola E, Mabro M, Artru P, Louvet C, de Gramont A. Oxaliplatin reintroduction in patients previously treated with leucovorin, fluorouracil and oxaliplatin for metastatic colorectal cancer. Ann Oncol. 2004;15:1210-4.

CONFIDENTIAL

This document is confidential. Do not disclose or use except as authorized.

(Mandalà 2009) Mandalà M, Barni S, Floriani I, Isa L, Fornarini G, Marangolo M, Mosconi S, Corsi D, Rulli E, Frontini L, Cortesi E, Zaniboni A, Aglietta M, Labianca R. Incidence and clinical implications of venous thromboembolism in advanced colorectal cancer patients: the 'GISCAD-alternating schedule' study findings. Eur J Cancer. 2009;45:65-73.

(Mass 2005) Mass RD, Sarkar S, Holden SN, Hurwitz H. Clinical benefit from bevacizumab (BV) in responding (R) and non-responding (NR) patients (pts) with metastatic colorectal cancer (mCRC) 2005 ASCO Annual Meeting Abstract No: 3514.

(Meyerhardt 2007) Meyerhardt JA, Stuart K, Fuchs CS et al. Phase II study of FOLFOX, bevacizumab and erlotinib as first-line therapy for patients with metastatic colorectal cancer. Ann Oncol. 2007;18:1185-9.

(Mick 2000) Mick R, Crowley JJ, Carroll RJ. Phase II clinical trial design for noncytotoxic anticancer agents for which time to disease progression is the primary endpoint. Control Clin Trials. 2000;21:343-59.

(Saltz 2007) Saltz LB, Lenz HJ, Kindler HL et al. Randomized phase II trial of cetuximab, bevacizumab, and irinotecan compared with cetuximab and bevacizumab alone in irinotecan-refractory colorectal cancer: the BOND-2 study. J Clin Oncol. 2007;25:4557-61.

(Tang 2007) Tang PA, Bentzen SM, Chen EX. Surrogate end points for median overall survival in metastatic colorectal cancer: literature-based analysis from 39 randomized controlled trials of first-line chemotherapy. J Clin Oncol. 2007;25:4562-8.

(Van Cutsem 2009) Van Cutsem E, Köhne CH, Hitre E, Zaluski J, Chang Chien CR, Makhson A, D'Haens G, Pintér T, Lim R, Bodoky G, Roh JK, Folprecht G, Ruff P, Stroh C, Tejpar S, Schlichting M, Nippgen J, Rougier P. Cetuximab and chemotherapy as initial treatment for metastatic colorectal cancer. N Engl J Med. 2009;360:1408-17.

(Von Hoff 1998) Von Hoff DD. There are no bad anticancer agents, only bad clinical trial designs--twenty-first Richard and Hinda Rosenthal Foundation Award Lecture. Clin Cancer Res. 1998;4:1079-86.

(Boellaard et al 2010) Boellaard R, O'Doherty MJ, Weber WA, et al. FDG PET and PET/CT: EANM procedure guidelines for tumour PET imaging: version 1.0. Eur J Nucl Med Mol Imaging. 2010 Jan;37(1):181-200.

This document is confidential. Do not disclose or use except as authorized.

23. Appendices

23.1 Performance status criteria

ECOG Performance Status Scale		Karnofsky Performance Scale	
Grade	Description	Percent	Description
0	Normal activity. Fully active, able to carry on all pre-disease performance without restriction.	100	Normal, no complaints, no evidence of disease.
		90	Able to carry on normal activity; minor signs or symptoms of disease.
1	Symptoms, but ambulatory. Restricted in physically strenuous activity, but ambulatory and able to carry out work of a light or sedentary nature (e.g., light housework, office work).	80	Normal activity with effort; some signs or symptoms of disease.
		70	Cares for self, unable to carry on normal activity or to do active work.
2	In bed < 50% of the time. Ambulatory and capable of all self-care, but unable to carry out any work activities. Up and about more than 50% of waking hours.	60	Requires occasional assistance, but is able to care for most of his/her needs.
		50	Requires considerable assistance and frequent medical care.
3	In bed >50% of the time. Capable of only limited self-care, confined to bed or chair more than 50% of waking hours.	40	Disabled, requires special care and assistance.
		30	Severely disabled, hospitalization indicated. Death not imminent.
4	100% bedridden. Completely disabled. Cannot carry on any self-care. Totally confined to bed or chair.	20	Very sick, hospitalization indicated. Death not imminent.
		10	Moribund, fatal processes progressing rapidly.

This document is confidential. Do not disclose or use except as authorized.

5	Dead.	0	Dead.
